# The complex regulation of competence in *Staphylococcus aureus* under microaerobic conditions

**DOI:** 10.1038/s42003-023-04892-1

**Published:** 2023-05-12

**Authors:** Shi Yuan Feng, Yolande Hauck, Fedy Morgene, Roza Mohammedi, Nicolas Mirouze

**Affiliations:** Université Paris-Saclay, CEA, CNRS, Institute for Integrative Biology of the Cell (I2BC), 91198 Gif-Sur-Yvette, France

**Keywords:** Pathogens, Bacterial genetics, Gene expression, Transcriptomics

## Abstract

To perform natural transformation, one of the three main Horizontal Gene Transfer mechanisms, bacteria need to enter a physiological differentiated state called genetic competence. Interestingly, new bacteria displaying such aptitude are often discovered, and one of the latest is the human pathogen *Staphylococcus aureus*.

Here, we show an optimized protocol, based on planktonic cells cultures, leading to a large percentage of the population activating the development of competence and a significant improvement of *S. aureus* natural transformation efficiencies. Taking advantage of these conditions, we perform transcriptomics analyses to characterize the regulon of each central competence regulator. SigH and ComK1 are both found essential for activating natural transformation genes but also important for activation or repression of peripheral functions. Even though ComK2 is not found important for the control of transformation genes, its regulon shows an important overlap with that of SigH and ComK1. Finally, we propose that microaerobic conditions, sensed by the SrrAB two-component system, are key to activate competence in *S. aureus*.

## Abstract (web summary)

Transcriptomic analysis identifies SigH and ComK1 as the key competence genes in *S. aureus*. In addition, oxygen influence on competence is studied.

## Introduction

A highly adaptive commensal organism, such as *Staphylococcus aureus*, possesses an array of genes that allows the bacterium to grow, infect and survive in a wide variety of ecological niches. The anterior nares are generally considered the native ecological niche of *S. aureus*, though the bacterium can be isolated from other areas of the human body, including the skin, the axillae, the groin, and the gastrointestinal tract^1^. While colonization is typically not harmful, *S. aureus* may breach innate host defenses and gain access to deeper tissues, causing a variety of superficial and invasive infections^2^. Furthermore, as a facultative anaerobe, *S. aureus* has the ability to grow and thwart the host immune system in the presence or absence of oxygen. This capacity is particularly important for *S. aureus* as its environment is known to be or become anaerobic during the course of an infection^3,4^.

In addition to these remarkable adaptive powers, *S. aureus* also became one of the most feared pathogens in the hospital because of the widespread emergence of antibiotic multi-resistant strains^5,6^. Horizontal gene transfer (HGT) of antibiotic resistance genes from other *S. aureus* strains or even from other genera was though for years to be exclusively mediated through conjugation and transduction^7^. However, a few years ago, the demonstration that *S. aureus* is capable of becoming naturally competent for genetic transformation^8^ changed our way of apprehending HGT in this important human pathogen.

Competence is a physiological adaptation that some bacterial species develop in response to various environmental signals^9^. In response to these stimuli, bacterial cells trigger signal transduction pathways, ultimately activating central competence regulators. All these steps are controlled by the so-called early competence genes. Interestingly, central competence regulators have been identified in several model organisms as transcriptional activators^10,11^ or alternative sigma factors^12^. Once activated, they initiate the expression of the late competence genes, among which are found all the genes essential for genetic transformation.

Importantly, three potential central competence regulators have been identified in *S. aureus*: the alternative sigma factor, SigH^13^, and two transcriptional regulators, ComK1 and ComK2^14^. In addition, it has been shown that *S. aureus* is able to naturally induce competence in a chemically defined medium called CS2^8^. The authors detected a maximum of 1.6% of competence-inducing cells after 8 h of growth in the CS2 medium, leading to transformation efficiencies that barely reached the detection limit (around 10^−10^) for a wild-type strain^8^.

In this study, our main objective was to characterize the development of competence in the human pathogen *S. aureus*. Our goal was to investigate all the steps involved, from the environmental stimuli sensed by the cells and the signal transduction pathways to the central competence regulators and their regulons. To reach such goals, we first developed a protocol to optimize the development of competence and genetic transformation in *S. aureus* planktonic cultures. Then, using various reporter strains and global transcriptomic analysis, we analyzed the role of the three central regulators during the development of competence in *S. aureus*. We particularly showed that while SigH and ComK1 are both essential for the expression of the genes involved in genetic transformation, the three regulators (SigH, ComK1, and ComK2) are all required for the full development of the competence transcriptional program. Finally, we propose that oxygen limitation, sensed by the SrrAB two-component system (TCS), probably activating SigH, controls the development of competence in the human pathogen *S. aureus*.

## Results

### An optimized protocol to induce competence for genetic transformation in *S. aureus* with high efficiency

**Fig. 1:**
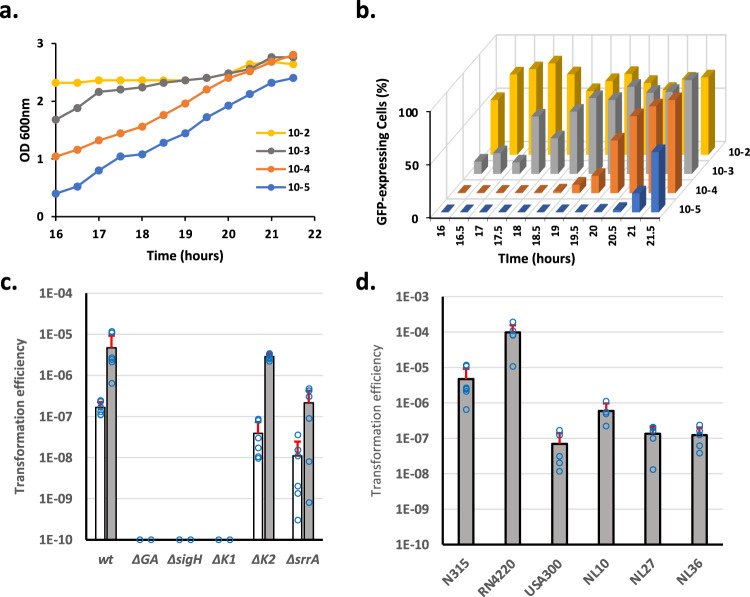
Competence for genetic transformation naturally develops with high efficiency in CS2 medium. **a** Growth of a wild-type strain expressing *gfp* under the control of the *comG* promoter (St29) in CS2 medium, between 16 and 21.5 h. Dilutions from 10^−2^ to 10^−5^ are shown. The 10^−2^ dilution was already in the stationary phase, while the 10^−3^ dilution was entering the stationary phase after 16 h of growth. The 10^−4^ and 10^−5^ dilutions were, respectively, in the late and early exponential phase at the beginning of the experiment. All the dilutions reached a similar final OD between 2.4 and 2.6. **b** The percentage of competent cells was measured using flow cytometry by analyzing the percentage of cells expressing GFP under the control of the *comG* promoter (P_*comG*_). In each diluted culture presented in panel a), the percentage of competent GFP-expressing cells increased in the late exponential phase and reached a maximum at the entry in the stationary phase. This graph shows a representative experiment that has been repeated three times (biological replicates, see Supplementary Fig. 2a). **c** Transformation efficiencies of wild type (N315ex w/o Phi), *comGA* (St137), *sigH* (St45), *comK1* (St37), *comK2* (St38), and *srrA* (St117) mutant strains using plasmidic (pCN34, Kan^R^, white bars) or chromosomal DNA (gray bars) (see Material and methods for details). Results are presented as mean ± SD. For each strain, the experiment was repeated at least five times (biological replicates). Individual experiments are shown as blue circles. **d** Transformation efficiencies of laboratory strains (N315, RN4220, and USA300) and MRSA (NL10, NL27) or MSSA (NL36) clinical isolates using chromosomal DNA (gray bars) (see material and methods for details). Results are presented as mean ± SD. For each strain, the experiment was repeated at least five times (biological replicates). Individual experiments are shown as blue circles.

We first decided to optimize the protocol published by Morikawa and his colleagues back in 2012^8^ (see Material and methods for details). In order to compare our results, we used the same reporter strain, expressing the *gfp* gene under the control of the *comG* late competence operon’s promoter (P_*comG*_*-gfp*)^8^. Briefly, the reporter strain was first streaked on a BHI agarose plate. Isolated colonies are then used to inoculate a pre-culture in the BHI medium. This pre-culture was then quickly stopped in exponential growth, centrifuged, washed, and used to inoculate a fresh culture in a CS2 medium. From this initial culture in fresh CS2, tenfold serial dilutions were made in closed Falcon tubes. Finally, cell density (OD600 nm) and the percentage of competent GFP-expressing cells were measured through flow cytometry in each diluted culture every 30 min. Interestingly, Fig. 1a, b demonstrates how our optimized protocol was able to induce the development of competence in up to 70% of the population in a given experiment (in reality, statistical analysis evaluated this percentage at 52 ± 15% of the population, *n* = 12). This percentage, more than ten times higher than in the literature, was previously calculated through the detection of GFP-expressing cells by microscopy^8^. Therefore, we verified that measurements performed by flow cytometry would not introduce any bias and provide similar results to microscopy (Supplementary Fig. 1).

We then confirmed that our optimized protocol, associated with improved development of competence, also led to higher transformation efficiencies. Interestingly, under laboratory conditions, an N315 wild-type strain reached a transformation efficiency around 1.5 × 10^−7^ (±0.6 × 10^−8^) with a replicative plasmid as donor DNA (Fig. 1c). Such a result is 1000 times higher than what has been previously published^8^. Importantly, the transformation efficiency even reached 4.2 × 10^−6^ (±4.1 × 10^−6^) when we used an *S. aureus* strain’ chromosome (harboring an antibiotic marker, see Material and methods) as exogenous DNA (Fig. 1c). To definitely confirm that the colonies growing in our assays were real transformants, we also showed that no transformation events could be detected using a strain in which *comGA*, an essential gene for genetic transformation^15^, was deleted (Fig. 1c).

Importantly, the protocol described here and optimized for the N315 strain also leads other laboratory strains (RN4220 and USA300) and clinical isolates (NL10, NL27, and NL36) to genetic transformation (Fig. 1d). Interestingly, RN4220 displayed very high transformation efficiencies (reaching 9.7 × 10^−5^ ±5.9 × 10^−5^) while USA300 (7 × 10^−8^ ±6.9 × 10^−8^) and the clinical isolates (NL10, 1.2 × 10^−7^ ±3.79 × 10^−7^; NL27, 1.6 × 10^−7^ ±4.8 × 10^−7^ and NL36, 1.2 × 10^−7^ ±7.9 × 10^−7^) showed lower numbers than N315. These results clearly demonstrate the robustness of our protocol, but also that further optimization might be required for new strains.

### Competence develops at a specific cell density and growth phase in CS2

Figure 1 clearly shows how competence was induced in each diluted culture using our optimized protocol. Each additional dilution was characterized by a 2-h delay in growth (Fig. 1a). Indeed, the 10^−5^ dilution needs more time to reach the stationary phase than the 10^−4^ dilution, which needs more time than the 10^−3^ dilution. Accordingly, the development of competence was also delayed in each consecutive diluted culture (Fig. 1b). However, GFP-expressing competent cells always appeared when the cultures approached OD = 2 and reached a maximum once each culture entered the stationary phase (Fig. 1a, b).

To further verify the existence of a correlation between competence development and cell density, we repeated this experiment three times (Supplementary Fig. 2a). This correlation clearly showed that competence development in *S. aureus* grown in CS2 reached a maximum as the cultures entered the stationary phase with an OD_600nm_ around 2.4 (in fact between 2.2 and 2.6) (Supplementary Fig. 2a). High cell density, sensed by bacterial cells through quorum sensing (QS), has been proposed and verified in several model organisms as important stress, inducing competence^16–18^. Therefore, we then hypothesized that a QS system could be involved in the development of competence in *S. aureus*. Interestingly, two QS systems have been identified in *S. aureus* (Agr and Lux^19^). Thus, we finally investigated the impact of *agrA* (encoding the Agr system transcriptional regulator,^19^) and *luxS* (encoding the Lux system regulator,^19^) gene deletion on the development of competence. Surprisingly, none of these genes were found involved in the induction of the *comG* operon’s expression (Supplementary Fig. 2b). This result seemed surprising, especially when compared to recent data where the deletion of *agrAC* decreased competence by a threefold factor^20^. Even though the protocols used in this study are different from ours, further investigations will be required to understand why QS does not seem involved in all conditions.

### P_*comG*_, P_*ssb*_, P_*comC*_, and P_*comF*_ are not controlled by the same regulators

As mentioned in the introduction, three potential competence regulators have been identified in the literature. Even though SigH has been shown to be important to activate the expression from P_*comG*_^8^, no role has been clearly assigned to ComK1 and ComK2. Here, using our optimized protocol, we decided to test the effect of *sigH*, *comK1*, and *comK2* deletion on the expression from four promoters: P_*comG*_ (a promoter only activated by SigH overexpression,^8,14^), P_*ssb*_ (a promoter that could only be activated by ComK1 over-expression,^14^), P_*comC*_ and P_*comF*_ (two promoters that could not be activated by any regulator^14^).

**Fig. 2:**
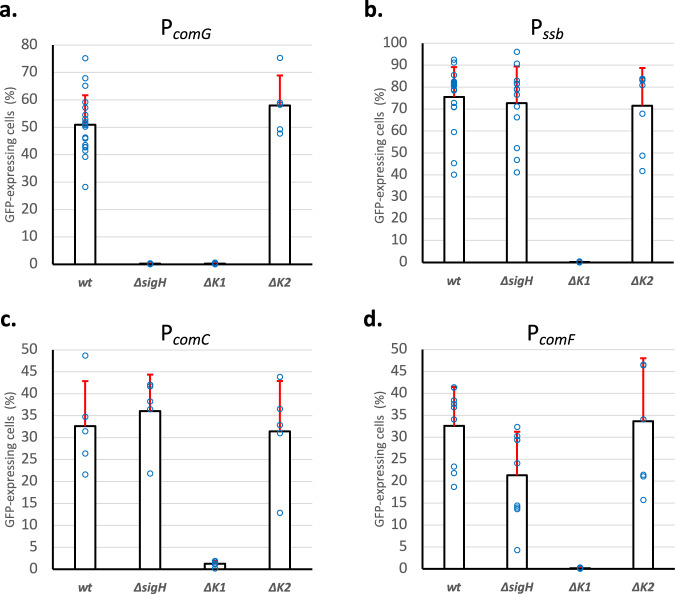
Expression of various genetic transformation-related genes is controlled by different central competence regulators. Percentage of the population expressing GFP under control of P_*comG*_ (St29, St51, St40, and St 41) (**a**), P_*ssb*_ (St50, St61, St64, and St67) (**b**), P_c*omC*_ (St48, St60, St63, and St66) (**c**), and P_*comF*_ (St233, St235, St234, and St236) (**d**) in a wild-type background or in the absence of *sigH*, *comK1*, or *comK2* was determined after 21 h of growth in CS2 medium. Results are presented as mean ± SD. Each experiment has been repeated at least five times (biological replicates). Individual experiments are shown as blue circles.

As expected, in a wild-type background, the four promoters were found activated with an average percentage of GFP-expressing cells of 51.87% for P_*comG*_, 77.26% for P_*ssb*_, 38.32% for P_*comC*_ and 38.56% for P_*comF*_ (Fig. 2 and Supplementary Fig. 3). When *sigH* was deleted, only the expression from P_*comG*_ was lost (Fig. 2a). This result confirms that SigH does not control the expression of all the genetic transformation genes and that at least one additional regulator must be involved. Interestingly, when *comK1* was inactivated, expression from all the promoters was abolished (Fig. 2a–d). Therefore, ComK1 is essential, alongside SigH, for the *comG* operon expression but is also absolutely required, alone, for *ssb* and *comC* expression. Interestingly, *comF* regulation seemed intermediate, as deletion of *comK1* abolishes *comF* expression while the absence of *sigH* decreases it by almost a twofold factor (Fig. 2d). Finally, in a *comk2* mutant, no effect could be detected for all promoters.

### SigH and ComK1 control genetic transformation, not ComK2

To complete our results (Fig. 2) and exhaustively characterize the SigH, ComK1, and ComK2 regulons, we then performed a global transcriptional analysis through RNA-sequencing by comparing the impact of the deletion of the genes encoding these three individual regulators on the competence transcriptional program.

**Fig. 3:**
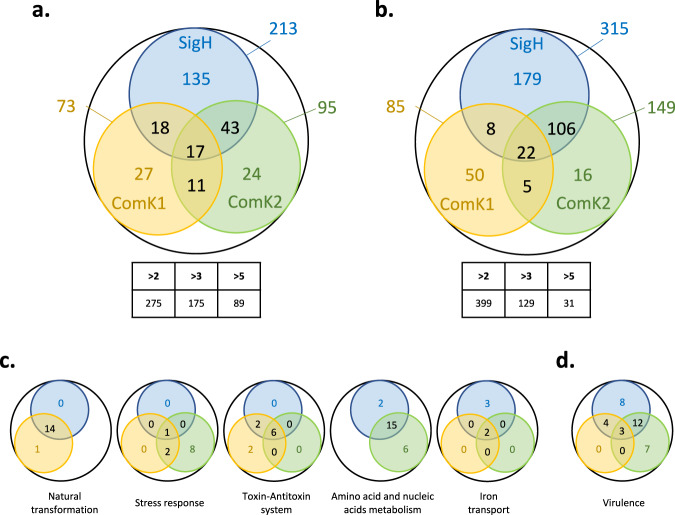
Global transcriptomic analysis (RNA-sequencing) of competence development in *S. aureus*. Late competence genes for which the expression is induced (>2-fold, **a**) or repressed (>2-fold, **b**). Each colored circle represents a regulon, for which the expression is activated or inhibited by SigH (in blue), ComK1 (in yellow), ComK2 (in green), or by the three (outside the black circle). The number of genes in each category is shown inside the circles or at the intersection between circles when the same gene is controlled by more than one regulator. The table below shows the total number of genes for which the expression is induced or repressed by a twofold, threefold, or fivefold factor. Activated (**c**) and repressed (**d**) late competence genes (>2-fold) are also presented per function. The number of genes controlled by each central competence regulator (SigH in blue, ComK1 in yellow, and ComK2 in green) is shown inside the circles or at the intersection between circles when the same gene is controlled by more than one regulator.

First, we focused on the genes involved in natural genetic transformation (Fig. 3 and Supplementary Table 1). The results previously obtained were confirmed despite some small differences (discussed in Supplementary Note 1). Overall, SigH and ComK1 were both found essential for the expression of most genetic transformation genes, with the exception of *ssb*, which is only controlled by ComK1. Again, no role could be attributed to ComK2. Finally, we verified the transformation efficiency of strains were the genes encoding the central competence regulators were individually deleted (Fig. 1c). Expectedly, only the absence of *sigH* or *comK1* abolished genetic transformation, while a *comK2* mutant displayed a transformation efficiency comparable to that of a wild type strain (Fig. 1c).

### SigH, ComK1, and ComK2 are all required for the full development of competence

In other model organisms, the competence transcriptional program always encompasses more genes than just the genes involved in genetic transformation^21–24^. It is clearly also the case in *S. aureus*. Indeed, in comparison to the *sigH*, *comK1*, and *comK2* mutant strains, 213/73/95 genes were respectively found overexpressed by at least a twofold factor in the wild-type strain (Fig. 3a). Furthermore, if we considered higher over-expression cutoffs, the number of genes found overexpressed were still important, with 84/38/53 genes overexpressed by a three-fold factor and even 35/23/31 genes overexpressed by a five-fold factor. The numbers found here, especially for the three-fold expression factor, are quite similar to what has been described in another model organisms^21–24^, revealing a true core set of around 100 late competence genes in *S. aureus*.

We then analyzed the functions associated to the genes induced during *S. aureus* competence transcriptional program (Fig. 3c, Supplementary Tables 2–4). In addition to the natural genetic transformation genes, we found genes involved in (i) the regulation of the stress response (mainly but not exclusively controlled by ComK2), (ii) genes encoding multiple putative Toxin/Antitoxin systems^25^ (controlled by the three regulators), (iii) genes involved in the amino and nucleic acids metabolism (controlled by SigH and ComK2) and (iv) genes involved in iron transport (mainly but not exclusively controlled by SigH). Importantly, our results clearly established that even though only SigH and ComK1 are essential for the expression of the genetic transformation genes, the central competence regulators (i.e., SigH, ComK1, and ComK2) are all absolutely required for the full development of the competence transcriptional program in *S. aureus*.

### Competence development is associated with virulence inhibition

Our global transcriptional analysis also revealed that the expression of numerous genes was inhibited during the development of competence in *S. aureus*. Indeed, we found that a total of 399 genes were inhibited (>2-fold) in the wild-type strain compared to the *sigH*, *comK1*, or *comK2* individual mutant strains (Fig. 3b). This represents more than 15% of all the genes present in the N315 *S. aureus’* genome.

Interestingly, more than 10% of the repressed genes (37 to be exact) are directly involved in virulence or virulence regulation (Fig. 3d, Supplementary Table 5). Among these genes were found many well-characterized virulence factors, including genes encoding for the capsule (CapA-O), serine proteases (SplA-F, SspA-C), intercellular adhesins (IcaAB), exoproteins (Hlg, Coa), surface proteins (ClfAB, fnb, FnbB, geh, sdrCD, Spa) and a lipase (Geh). In addition, the expression of an important TCS known to drive the expression of over 20 virulence factors in vivo, *saeRS*^26^, was found repressed during competence. Even though all the central competence regulators were found involved in virulence repression, SigH and ComK2 played a major role with an important overlap between the genes they each repress (Fig. 3d, Supplementary Table 5). Unfortunately, a comparison of wild type and *sigH* or *comK2* mutant exoproteomes did not show any significant difference in the number of virulence factors (Supplementary Fig. 4). Importantly, in our cultures, two populations co-exist, competent and non-competent cells, each representing 50% of the total population. Therefore, as the non-competent cells still produce virulence factors, the effect of the inhibition associated with the competent cells on the total amount of virulence factors produced in the culture would be underestimated. Alternatively, the cells could have been collected at a time point that might not be appropriate to observe the effect on the exoproteome.

### Oxygen limitation is an important environmental signal to induce the development of competence

**Fig. 4:**
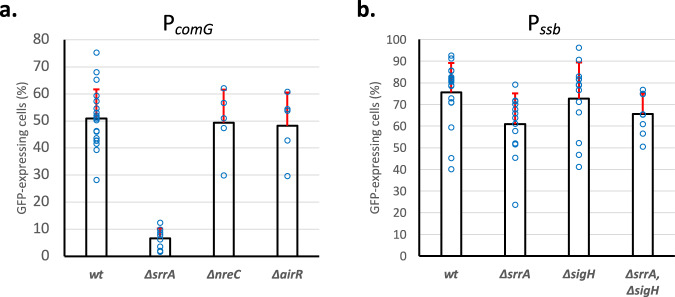
The SrrAB two-component-system controls the development of competence in *S. aureus*. **a** Percentage of cells expressing GFP from P*comG* in wild type (St29), *ssrA* (St145), *nreC* (St158), and *airR* (St177) mutant strains. Results are presented as mean ± SD. Each experiment has been repeated at least five times (biological replicates). Individual experiments are shown as blue circles. **b** Percentage of cells expressing GFP from P*ssb* in wild type (St50), *srrA* (St147), *sigH* (St61), and *srrA*/*sigH* double mutant (St252) strains. Results are presented as mean ± SD. Each experiment has been repeated at least five times (biological replicates). Individual experiments are shown as blue circles.

Finally, competence development in *S. aureus* has been demonstrated in different conditions associated to different oxygen availability^8^. Thus, we wondered if oxygen-sensing TCS present in *S. aureus* (i.e., SrrAB^27^, NreBC^28^, and AirRS^29^) were involved during the early competence regulation steps in the course of planktonic growth in the CS2 medium. To test such a hypothesis, we compared the expression from the *comG* promoter in wild-type and *srrA*, *nreC*, and *airR* mutant strains. In this experiment, deletion of *nreC* and *airR* did not affect the expression from the *comG* promoter (Fig. 4a). However, when *srrA* was absent, *comG* expression was decreased by a sevenfold factor (Fig. 4a). Since both SigH and ComK1 are involved in the *comG* operon expression (Fig. 2a), we then decided to determine which central competence regulator was under the control of SrrAB. As *ssb* expression was found only controlled by ComK1 (Fig. 2b), we finally tested the effect of *srrA* deletion on its expression. In the absence of *srrA*, the expression of *ssb* was not affected (Fig. 4b). In addition, deletion of both *sigH* and *srrA* had the same impact on the *ssb* expression as the individual *sigH* and *srrA* mutant strains (Fig. 4b). Altogether, these results suggested that SrrAB might activate SigH to control the expression from the *comG* promoter. Moreover, the implication of SrrAB in the regulatory pathways leading to competence development and genetic transformation implies that the absence of *srrA* must have an effect on *S. aureus* genetic transformation efficiencies. Indeed, when the *srrA* gene was deleted, a 15 and 19-fold decrease (respectively obtained using plasmid or chromosomal DNA) in *S. aureus* genetic transformation efficiency was observed (Fig. 1c).

**Fig. 5:**
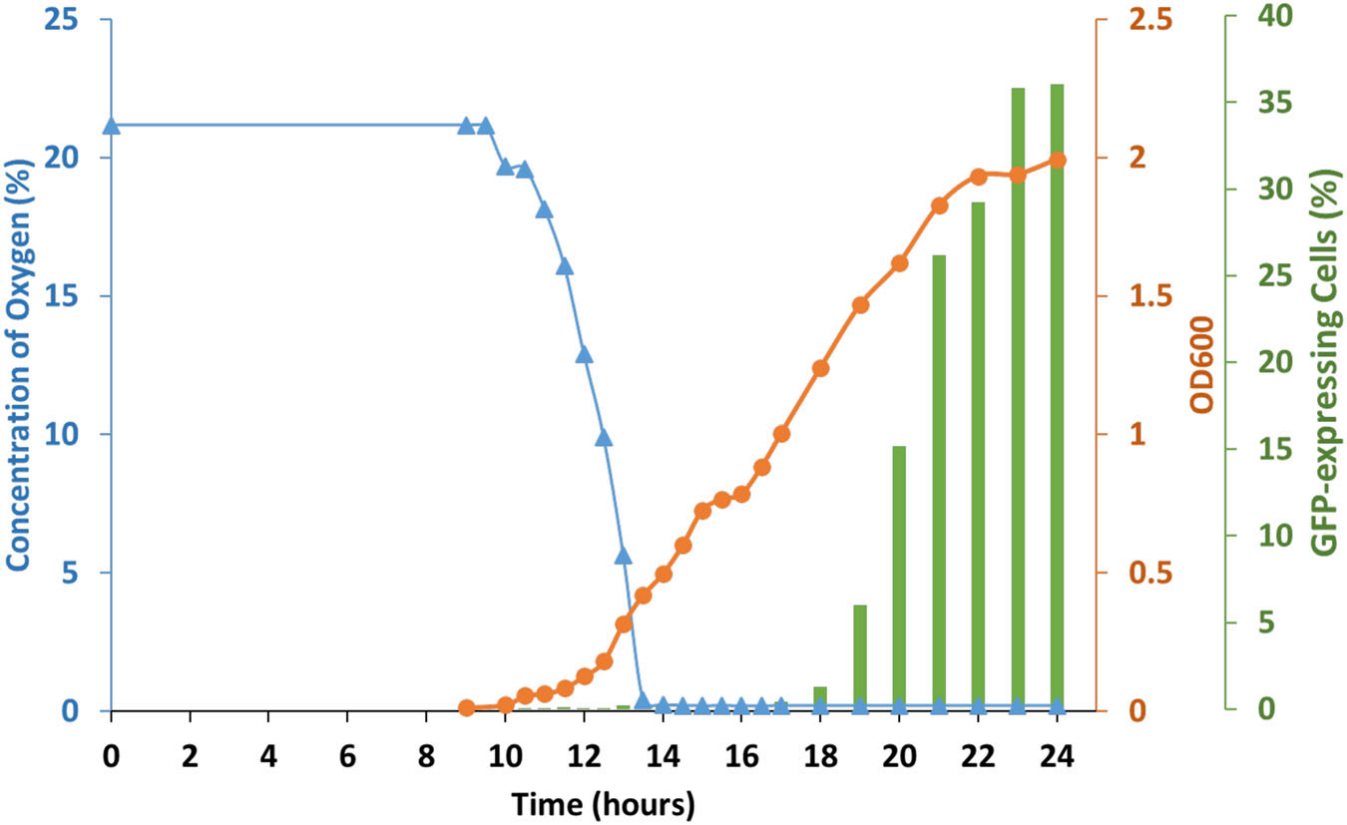
Growth in CS2 medium leads to microaerobic conditions. A wild-type strain (St29) expressing GFP under the control of P_*comG*_ was grown in a CS2 medium for 24 h. When growth became detectable, the oxygen concentration, the percentage of GFP-expressing cells, and OD600 nm were measured every 30 min. As the OD increased, the concentration of oxygen quickly dropped from 21% to 0.27%, while the OD only reached 0.5. Importantly, our assay allows us to affirm that complete anaerobic conditions are not reached as our sensors are able to measure with confidence oxygen concentrations that are ten times lower. A few minutes later, the growth reproducibly marked a pause, probably necessary for the cells to adapt to low oxygen conditions, which in turn induced an increase in the percentage of competent GFP-expressing cells. This Figure shows a representative experiment that has been reproduced five times (see Supplementary Fig. 5 for replicates).

Oxygen-sensing TCS allows *S. aureus* to survey and respond to microaerobic or anaerobic conditions. In particular, SrrAB has been shown to be a global regulator of virulence factors under low-oxygen conditions^27^. It is, therefore, tempting to speculate that during *S. aureus* growth in the CS2 medium, the oxygen concentration dropped in the culture. To test this hypothesis, we finally measured the concentration of dissolved oxygen throughout growth in CS2 (Fig. 5 and Supplementary Fig. 5). Interestingly, as the OD started to increase, the oxygen concentration quickly dropped. Indeed, while the oxygen concentration stayed constant at 21% for the first 8 h, it then decreased down to 0.27% during the next 6 h, while the culture’s OD only reached 0.5. A few minutes after the oxygen concentration reached its lowest point, the culture reproducibly paused for roughly 1 h, potentially to adapt to these new low oxygen conditions (Fig. 5). Finally, following the drop in the oxygen concentration and the pause in growth, GFP-expressing competent cells started to emerge (Fig. 5). Therefore, we can confidently propose that using our optimized protocol, growth in CS2 medium is associated to a quick limitation in the oxygen availability, leading to microaerobic conditions sensed by SrrAB which in turn activates SigH for the induction of competence in *S. aureus*.

## Discussion

Previously, the work of Fagerlund and her colleagues^14^ clearly established that the central competence regulator’s individual or combined over-expression was not enough to induce the full development of competence in *S. aureus*. In this study, we present an optimized protocol allowing the optimum induction of genetic competence in *S. aureus*. The such protocol was essential to lead the genetic study proposed here. Importantly, the resulting transformation efficiencies detected with several laboratory strains and clinical isolates ultimately demonstrate the true potential of this HGT mechanism to modulate *S. aureus* genetic plasticity and antibiotic resistance genes acquisition in vivo.

**Fig. 6:**
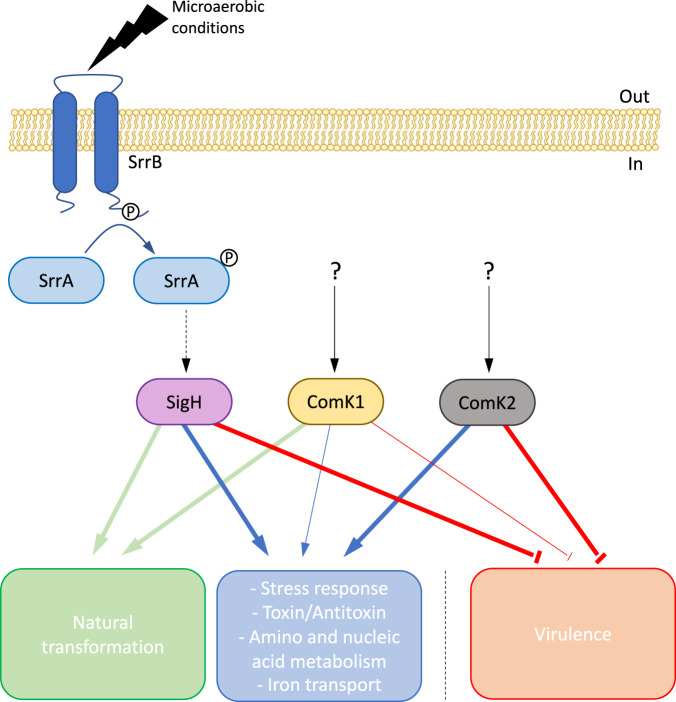
Model for the regulation of the natural development of competence in *S. aureus*. Three central competence regulators have been identified, namely SigH, ComK1, and ComK2^8,14^. All three regulators are essential for a complete development of the competence transcriptional program. While SigH and ComK1 are absolutely required for the expression of genetic transformation genes, ComK2 (alongside SigH and ComK1) is also essential for the induction of additional cellular functions (i.e., stress response, toxin/antitoxin systems, amino and nucleic acid metabolism, iron transport…). In addition, the development of competence is characterized by the inhibition of virulence, mainly controlled by SigH and ComK2. Finally, we have shown that the natural development of competence is induced as the oxygen concentration in the culture is drastically decreased. This oxygen rarefaction is probably sensed by the SrrAB two-component system, which in turn activates the central competence regulator SigH.

In addition, we also established that three central competence regulators are essential for a complete development of the competence transcriptional program in *S. aureus*. While the importance of SigH was already known, we demonstrate for the first time the essentiality of ComK1 for the expression of the genes involved in genetic transformation. Importantly, we also reveal how ComK2 is involved, alongside SigH and ComK1, in the complete development of the competence transcriptional program (Fig. 6). Indeed, our global transcriptomic study clearly shows that in addition to genetic transformation, numerous other functions are also induced during competence, a feature shared with other model organisms. It will be important to investigate in the future how these other biological processes (i.e., stress response^30^, amino^31^ and nucleic^32^ acid metabolism or toxin/antitoxin systems^33,34^) participate to the establishment of the competence environmental adaptation.

The presence and involvement of three central competence regulators in *S. aureus*, each probably induced by distinct signal transduction pathways, is a striking feature. The overlapping of the SigH, ComK1, and ComK2 regulons is another one, even though such overlapping has already been described in *Vibrio cholerae*, in which two central competence regulators have been identified^35^. One hypothesis could be that *S. aureus* needs multiple regulatory pathways in order to integrate a wider range of cues to decide whether or not to become competent for genetic transformation. According to this hypothesis, several environmental signals should be present concomitantly to allow the optimum development of genetic competence in *S. aureus*. On the other hand, such complex global regulation provides multiple targets to modulate competence development when *S. aureus* is not in these optimal conditions. Interestingly, ComK2, which does not control the expression of the genetic transformation genes in microaerobic environments, could become involved in response to different specific environmental signals, complexifying the regulation for the development of competence in *S. aureus*.

Furthermore, it is interesting to mention that the regulation of competence development in *S. aureus* shares traits with several important historical model organisms: *S. pneumoniae* and its alternative sigma factor (ComX), phylogenetically close to SigH^13^, *B. subtilis* and its central competence regulator, ComK, homologous to ComK1 and ComK2^14^ and *V. cholerae* through the presence of several central competence regulators^18^. Thus, it would be interesting and probably important to test in the future if other regulatory features present in these historical model organisms could also be used by *S. aureus* to control or modulate the development of competence.

Finally, we demonstrate how oxygen limitation, leading to microaerobic conditions sensed by the SrrAB TCS, controls the development of competence in *S. aureus*. Since our cultures are performed in closed tubes, similarly to what was previously published^36^, we think that growing *S. aureus* in CS2 leads to rapid consumption of dissolved oxygen that cannot be replaced by atmospheric oxygen. Results from the literature already indicated that *S. aureus* had the ability to induce competence under different oxygen concentrations^8,20^. Induction of competence under anaerobic conditions^8^, during biofilm formation^20^ or in response to ROS^37^, further reinforces this idea. We also proposed that SrrAB could activate SigH (through transcription, translation, or stability) in response to variations in the environmental oxygen concentration. Additional work will be required to fully understand how a decrease in the oxygen concentration activates, directly or indirectly, SigH through SrrAB. Interestingly, the work of Cordero and colleagues shows how competence development is associated with dramatic changes in carbon metabolism^37^. It would be, therefore, important to test if the regulation of the cellular physiology, in response to oxygen limitations, could be involved in the development of competence in *S. aureus*.

Importantly, microaerobic conditions are often encountered by *S. aureus* in vivo. Indeed, during an infection, energy-consuming activated neutrophils trigger oxygen deficiency, while macrophages, dendritic cells, and T cells induce inflammation, altering blood flow to tissues and reducing oxygen levels dramatically^38^. In addition, oxygen-restricted microenvironments are formed during biofilm-associated infections^39^ or abscesses^40^. Therefore, during the course of an infection, *S. aureus* is often exposed to the environmental conditions described in this manuscript and leading to the development of competence for genetic transformation, reinforcing the true potential of this HGT mode in vivo. However, our global transcriptomic analysis also revealed that the transcription of numerous virulence genes was repressed during the natural development of competence (Fig. 6). Therefore, as an in vivo model, we can propose that during the course of an infection, *S. aureus* cells induce the expression of the virulence regulon, which would ultimately lead to a local oxygen deficiency. A restricted percentage of the population then senses this environmental signal and, in response, induces competence for genetic transformation in a restricted part of the population. Ultimately, the competent *S. aureus* cells repress virulence and promote HGT, while the rest of the population continues to promote the infection.

## Materials and methods

### Bacterial strains and culture conditions

N315^8^, RN4220^41^, and USA300^42^ *S. aureus* strains, as well as clinical isolates^43^ used in this project, are all listed in Supplementary Table 6. *S. aureus* strains were grown in BHI medium (Becton, Dickinson, and Company) or a completely synthetic medium called CS2^8^, depending on the experiment. When necessary, antibiotics were used to select specific events (Kan, 200 µg/mL; Cm, 10 µg/mL).

### Optimized dilution protocol to naturally induce competence in *S. aureus*

Cells were isolated from −80 °C stock on the BHI plate. Four clones were inoculated in 10 mL of BHI and incubated at 37 °C with shaking at 180 rpm until OD reached 2.5. This pre-culture was then centrifuged, washed in fresh CS2 medium, and used to inoculate 10 mL of fresh CS2 medium (OD = 0.5) in closed 50 mL Falcon tubes. From this initial CS2 culture, serial 10-fold dilutions were performed to generate 10^−1^, 10^−2^, 10^−3^, 10^−4^, and 10^−5^ cultures closed 50 mL Falcon tubes, and more if needed. Several tubes for each dilution were prepared in order to take individual samples along growth (i.e., each Falcon tube was only opened once for one sample). Dilutions were finally incubated overnight at 37 °C with shaking at 120 rpm. Cells were collected when the OD was between 2 and 2.2 (GFP reporter strains, Figs. 2 and 3) or throughout growth (GFP reporter strains, Fig. 1, oxygen measurements, Fig. 4).

### Construction of GFP reporter plasmids and strains

Promoters of interest were inserted in the pRIT-GFP plasmid^44^ using the Gibson assembly method. The promoters and the pRIT-GFP plasmid were first amplified with dedicated primers. All the oligonucleotides used in this study are listed in Supplementary Table 7. In every primer’s tail, we designed 25–50 bp homologous overlap regions between the extremities of each promoter and the pRIT-GFP linear plasmid. The promoters and the pRIT-GFP plasmid were amplified using the Phusion High-Fidelity DNA Polymerase (purchased from Thermo Scientific). All the PCR fragments were then purified using a standard commercial silicon column (PCR cleanup kit, Macherey-Nagen) and verified by gel electrophoresis for the absence of non-specific or minor PCR fragments.

Gibson assembly master mix was prepared by adding 320 µL of 5× ISO buffer (25% w/v PEG-8000; 500 mM Tris-HCl, pH 7.5; 50 mM MgCl_2_; 50 mM DTT; 5 mM NAD; 1 mM each dNTP), 0.64 µL 10 U/µL T5 exonuclease, 20 µL of 2 U/µL Phusion polymerase, 160 µL of 40 U/µL Taq DNA ligase and 699.36 µL of water (all reagents were purchased from New England Biolabs). Five microlitres of the two DNA fragments mixture containing 100 ng of linear pRIT plasmid and a 3-fold excess of inserts were added to 15 µL of Gibson assembly master mix. The reaction tubes were then incubated at 50 °C for 1 hr. Finally, 1 µL of the assembly reaction was transformed into IM08B electro-competent *Escherichia coli* cells. Transformed *E. coli* cells were incubated at 37 °C on LB agar with 100 µg/mL ampicillin.

In order to select the transformants containing the expected plasmid, colony PCR was performed. The resulting plasmids were extracted from positive transformants (overnight cultures) and purified using a commercial kit (NucleoSpin Plasmid extraction kit, Macherey-Nagen). All the plasmids were verified by sequencing (GATC company). Finally, in order to obtain the final reporter strains, *S. aureus* electrocompetent cells were transformed with each constructed plasmid.

### Construction of *S. aureus* deletion mutants

In order to investigate the role of the main genes predicted to be involved in the regulation of competence development in *S. aureus*, listed in Table S1, allelic replacement constructs were cloned into the temperature sensitive pIMAY plasmid^44^. All the primers used for cloning in the present study are listed in Supplementary Table 7. Fragments corresponding to 1 kbp flanking regions of the genes to delete were amplified from *S. aureus* N315 genomic DNA using primers flanked by restriction enzyme sites compatible with the multiple cloning site (MCS) of pIMAY. The upstream or downstream regions were digested using the two chosen restriction enzymes and ligated into the pIMAY, opened with the same enzymes. The resulting plasmids were electro-transformed into the IM08B *E. coli* strain. Colony PCR was then used to verify the structure of the plasmids present in the transformants. Plasmids were purified from the positive colonies using a commercial kit (NucleoSpin Plasmid extraction kit, Macherey-Nagen). After verifying the sequence of the plasmid (GATC company), *S. aureus* N315ex woϕ strain was transformed by electroporation and plated on BHI agar supplemented with chloramphenicol (10 mg/mL) and incubated at 28 °C.

In order to allow pIMAY integration into the chromosome, a single colony from the transformation plate was resuspended in 200 µL of BHI. The suspension was diluted 10-fold down to 10^−3^, and 100 µL of each dilution was spread on BHI supplemented with chloramphenicol (10 mg/mL) and incubated at 37 °C overnight. The next day, colonies were subsequently streaked in the same conditions. Meanwhile, colony PCR analysis was performed to check the absence of extrachromosomal pIMAY and whether plasmid integration had occurred in the upstream or downstream region.

Based on the colony PCR results, an overnight culture in BHI at 28 °C without chloramphenicol was performed. The overnight culture was then diluted 10-fold down to 10^−7^. Hundred microlitres of 10^−4^ to 10^−7^ dilutions were plated onto BHI containing 1 µg/mL anhydrotetracycline (aTc). The plates were incubated at 28 °C for 2–3 days. Colonies were then patched on BHI (without antibiotic) and BHI supplemented with chloramphenicol (10 mg/mL) plates and grown at 37 °C overnight. Chloramphenicol-sensitive colonies were screened by colony PCR to identify clones containing the desired mutation. Mutant strains were finally verified by PCR and DNA sequencing.

### Flow cytometry to determine the percentage of the population expressing GFP

Following growth in CS2, 500 µL of cells were harvested by centrifugation at 11,000*g* for 1 min. Pellets were resuspended in 500 µL of cold 70% ethanol and incubated on ice for 20 min in order to fix the cells. Then, *S. aureus* cells were resuspended in 500 µL of PBS (pH 7,4) after centrifugation at 11,000*g* for 1 min. Finally, the percentage of the population expressing GFP was evaluated by Flow cytometry (Cytoflex top-bench cytometer, Beckman-Coulter). Following Forward- and Side-scatter detection to identify individual cells, a 488 nm laser was used to distinguish GFP-expressing competent cells by comparison with the auto-fluorescence of a strain that did not express GFP (St12) (see Supplementary Fig. 1a).

It is important to mention that GFP is a very stable protein. Therefore, once the maximum percentage of competent cells was reached, this number stayed constant for hours. This feature does not mean that competence stays ‘open’ for hours but rather that once the maximum is reached, no new competent cells appear.

### Microscopy to determine the percentage of GFP-expressing cells

Following growth in CS2, cells were harvested and treated as explained above (see flow cytometry). Fluorescence images of cells were taken using a confocal laser-scanning microscope (ImagerieGif platform). GFP was excited at 488 nm using the blue laser, and fluorescence images were collected using the green channel. Images were reconstituted using the ImageJ software.

### Genetic transformation of competent *S. aureus* cells

Wild-type strain (St12) as well as *comGA* (St137), *comK1* (St37), *comK2* (St38), and *sigH* (St45) mutant strains were first grown to competence using our optimized dilution protocol. We chose to perform the transformation experiments using the −2 dilution culture for each strain. Cells were naturally transformed following the protocol previously published^8^ with some adjustments. Briefly, at each time point (every half hour), 2 mL of cells were harvested by centrifugation at 10,000*g* for 1 min at 4 °C, resuspended in 2 mL of fresh CS2, and equally divided into two tubes. One or 5 µg of donor-DNA (plasmid or chromosome) was added to one of the tubes (the second tube is used as a “no DNA” control) and incubated at 37 °C for 2.5 h with agitation at 180 rpm. Ten or 100 µL (tube with DNA) or 1 mL (“no DNA control”) from each tube where finally mixed with 25 mL of melted BHI agar pre-cooled to 55 °C together with an antibiotic, and the mixture was poured into Petri dishes. After solidification, the plates were incubated at 37 °C for 48 h. At each time point, the viability was also evaluated by serial dilution on BHI agar plates. Transformation efficiencies were finally calculated by dividing the number of transformants detected in 1 mL of culture by the total number of cells in the same volume. Numbers presented in Fig. 1c represent the mean of the highest transformation efficiencies detected along growth during each experiment. The experiments have been repeated for each strain at least 5 times to provide strong statistical relevance.

### Donor DNA preparation

*Plasmid*. The pCN34 plasmid (Kan^45^) was used in some of the genetic transformation experiments (Fig. 1c). pCN34 was purified from the St197 strain. Briefly, 50 mL of culture were harvested by centrifugation and the plasmid was purified using a plasmid purification kit (Macherey-Nagen).

*Chromosomal DNA.* strain St294 was used to provide donor chromosomal DNA (Fig. 1c, d). In St294, the pIMAY-INT^14^ plasmid (Cm) was inserted in the chromosome at the INT chromosomal site^14^. The plasmid insertion was verified by PCR while no replicating plasmid could be detected. Briefly, 100 mL of culture were centrifuged and resuspended in 5 mL of TEG (Tris 5 mM, pH8; EDTA, 10 mM; Glucose, 1%) complemented with 500 µL of Proteinase K (10 mg/mL), 2 mL of lysis buffer (NaOH, 0.2 N; SDS, 1%) and 20 g of glass beads (Stratech, #11079-105, 0,5 mm in diameter). The cells were then broken using 5 cycles of vortex (1 min each) with 1 min in ice between each cycle. To finish cell lysis, 3 mL of lysis buffer were added for 5 min at room temperature and neutralized with 6 mL of NaAc (3 M, pH 4.8). Finally, chromosomal DNA present in the supernatant was precipitated using 96% ethanol (1 ml of EtOH for 500 µL of supernatant) after 2 hours of incubation at −20 °C. After centrifugation, chromosomal DNA was washed using 300 µL of cold 70% ethanol. Precipitated chromosomal DNA was finally resuspended in 300 µL of Tris 5 mM, pH8.

### RNA-sequencing

*Sampling and isolation*. Cultures of *S. aureus* were grown in CS2 medium at 37 °C and 180 rpm until the OD600 reached 2. To quench cellular metabolism / transcription and to stabilize RNAs, cells were harvested by centrifugation at 10,000*g* for 1 min at 4 °C and the pellets immediately frozen in liquid nitrogen before storage at −80 °C. Three independent biological replicates were collected for each of the four strains (wild type, St29; Δ*comK1*, St40; Δ*comK2*, St41; Δ*sigH*, St61). For extraction of RNA, cells were lysed using Lysing Matrix B and a FastPrep instrument (both MP Biomedicals), and RNA were isolated using the RNeasy Mini Kit (Qiagen). RNAs were treated with TURBO DNase (Ambion), purified using the RNA Cleanup protocol from the RNeasy Mini Kit (Qiagen), and stored at −80 °C. The integrity of the RNA was finally analyzed using an Agilent Bioanalyzer (Agilent Technologies).

*rRNA depletion, library construction and sequencing*. Removal of 23 S, 16 S, and 5 S rRNA using the RiboZero rRNA Removal Kit (Epicenter) (two times), strand-specific library construction yielding fragments of size range 100–500 bp, pooling of the 12 indexed libraries, sequencing in one flow-cell lane on a Illumina HiSeq2000 instrument with a 75 nt paired end protocol, and demultiplexing of the 12 samples of indexed reads was performed by the “Next Generation Sequencing (NGS) Core Facility” from the Institute for the Integrative Biology of the Cell (I2BC, Gif sur Yvette, France).

*Read mapping and analysis of differential expression*. Differential expression of all annotated features was assessed using the R statistical programming environment. Differential expression was determined between the wild-type strain samples (St29, *n* = 3) and each of the 9 samples (each *n* = 3) in which *sigH*, *comK1*, or *comK2* were absent. The output from the differential expression analysis is presented in Supplementary Tables 2–5. Genes with false discovery rate (FDR)-corrected *P*-values < 0.01 and a ratio of differential expression superior to 2, 3, or 5 were considered significantly differentially expressed and are presented.

*Data accessibility*. The whole set of RNA-seq data is compiled and accessible under the GEO submission GSE224932.

### *S. aureus* exoproteome analysis

*Exoprotein isolation*. *S. aureus* cultures (wild type, St12; Δ*sigH*, St45 and Δ*comK2*, St 38) were grown in 10 ml CS2 in 50 mL Falcon tubes for 19.5 h. Culture supernatants were collected by centrifugation at 6000 rpm for 10 min (4 °C) to remove bacteria, followed by filtration through a 0.22 µm filter to remove cell debris. The proteins in the culture supernatants were precipitated in 20% (v/v) trichloroacetic acid (TCA) at 4 °C overnight. The precipitated proteins were sedimented by centrifugation at 13000 rpm for 45 min (4 °C) and the pellets were washed with 96% ethanol. The protein pellets were finally centrifuged at 13,000 rpm for 30 min (4 °C), the remaining ethanol was removed and the pellets were allowed to air dry.

*Exoprotein profiling*. Precipitated exoproteins were resuspended in 18 µl of 1× PBS and incubated for 30 min at room temperature. After addition of 20 µl of 2× Tris-Glycine SDS Novex buffer (ThermoFisher) and 1 M DTT, the samples were incubated for 10 min at 95 °C. Exoproteins were finally separated in a 4–12% Tris-Glycin gel (Invitrogen) and visualized using Coomassie staining.

*Exoprotein sample preparation and LC-MS analysis*. The bands containing the whole sample were cut and subjected to in-gel trypsin digestion using standard conditions including reduction and alkylation. Trypsin-generated peptides were analyzed by nanoLC–MSMS using a nanoElute liquid chromatography system (Bruker) coupled to a timsTOF Pro mass spectrometer (Bruker). Peptides were loaded on an Aurora analytical column (ION OPTIK, 25 cm × 75 m, C18, 1.6 m) and separated with a gradient of 0–35% of solvent B for 100 min. Solvent A was 0.1% formic acid and 2% acetonitrile in water and solvent B was acetonitrile with 0.1% formic acid. MS and MS/MS spectra were recorded from m/z 100 to 1700 with a mobility scan range from 0.6 to 1.4 V s/cm^2^. MS/MS spectra were acquired with the PASEF (Parallel Accumulation Serial Fragmentation) ion mobility-based acquisition mode using a number of PASEF MS/MS scans set as 10.

*Data analysis*. MS and MSMS raw data were processed and converted into mgf files with DataAnalysis software (Bruker). Protein identifications were performed using the MASCOT search engine (Matrix Science, London, UK) against *S. aureus* database. Database searches were performed using trypsin cleavage specificity with two possible missed cleavages. Carbamidomethylation of cysteines was set as fixed modification and oxidation of methionines as variable modification. Peptide and fragment tolerances were set at 10 ppm and 0.05 Da, respectively. Proteins were validated when identified with at least two unique peptides. Only ions with a score higher than the identity threshold and a false-positive discovery rate of less than 1% (Mascot decoy option) were considered. Mass spectrometry based-quantification was performed by label-free quantification using spectral count method. Total MS/MS spectral count values were extracted from Scaffold software (version Scaffold 4.11.1, Proteome software Inc, Portland, OR) filtered with 95% probability and 0.1% FDR for protein and peptide thresholds, respectively. For statistical analysis, missing values occurring in spectral count datasets at protein-level were imputed by a constant value fixed at 0.1. In order to take into account within-sample variation in spectral count datasets, a beta-binomial test was performed based on triplicates MS/MS analyses with *P*-values calculated using R package ‘ibb’ (version 13.06, 61). Proteins were filtered on a *P*-value < 0.05 and a fold change larger than two.

The mass spectrometry proteomics data have been deposited to the ProteomeXchange Consortium via the PRIDE partner repository with the dataset identifier PXD040550 and 10.6019/PXD040550.

### Oxygen concentration measurements

Oxygen concentrations were measured using the SP-PSt3-SA23-D3-OIW oxygen sensor spots (PreSens GmbH, Regensburg, Germany). These sensor spots were attached to the inner wall of 50 mL Falcon tubes with silicone glue so that the spots would always be immerged during the experiments (i.e., below the 5 mL mark). The Falcon tubes were closed at T0 and remained closed for the entire experiment.

The sensor spots are covered with an oxygen-sensitive coating where molecular oxygen quenches the luminescence of an inert metal porphyrine complex immobilized in an oxygen-permeable matrix. This process guarantees a high temporal resolution and a measurement without drift or oxygen consumption.

The photoluminescence lifetime of the luminophore within the sensor spot was measured using a polymer optical fiber linked to an oxygen Meter (Fibox 4 trace; PreSens GmbH). Excitation light (505 nm) was supplied by a glass fiber, which also transported the emitted fluorescence signal (600 nm) back to the oxygen meter. Briefly, an oxygen measurement was realized, through the Falcon tube plastic, by simply approaching the optical fiber from the sensor spot. At each time point, the oxygen concentration was measured three times and the results provided represent the mean of these three measurements. In our experiments, the oxygen concentration was measured every 30 min.

### Reporting summary

Further information on research design is available in the Nature Portfolio Reporting Summary linked to this article.

## Supplementary information

Mirouze_Peer review file

Supplemental material

Description of Additional Supplementary Files

Supplementary Data

Reporting summary

## Data Availability

The whole set of RNA-seq data is compiled and accessible under the GEO submission GSE224932. The mass spectrometry proteomics data have been deposited to the ProteomeXchange Consortium via the PRIDE partner repository with the dataset identifier PXD040550 and 10.6019/PXD040550. All other data are available from the corresponding author on reasonable request. Source data is available as Supplementary Data table.
