# Supplementary material for: The complex regulation of competence in Staphylococcus aureus under microaerobic conditions
Source: Commun Biol. 2023 May 12;6:512. doi: 10.1038/s42003-023-04892-1 (PMC10182052; doi:10.1038/s42003-023-04892-1)
Supplement: Supplementary file 2 — Supplemental material [file 42003_2023_4892_MOESM2_ESM.pdf]

Supplementary Information for

The complex regulation of competence in *Staphylococcus aureus* under Microaerobic conditions

Shi Yuan Feng [‡,1], Yolande Hauck [‡,1], Fedy Morgene [1], Roza Mohammedi [1] and Nicolas Mirouze [*,1]

[1] Université Paris-Saclay, CEA, CNRS, Institute for Integrative Biology of the Cell (I2BC), 91198, Gif-sur-Yvette, France

[‡] Co-first authors
[*] Corresponding author
Email: nicolas.mirouze@i2bc.paris-saclay.fr

This PDF file includes:

Supplementary Figures 1 to 5
Supplementary Tables 1 to 7
Supplementary Note 1

Supplementary Figures

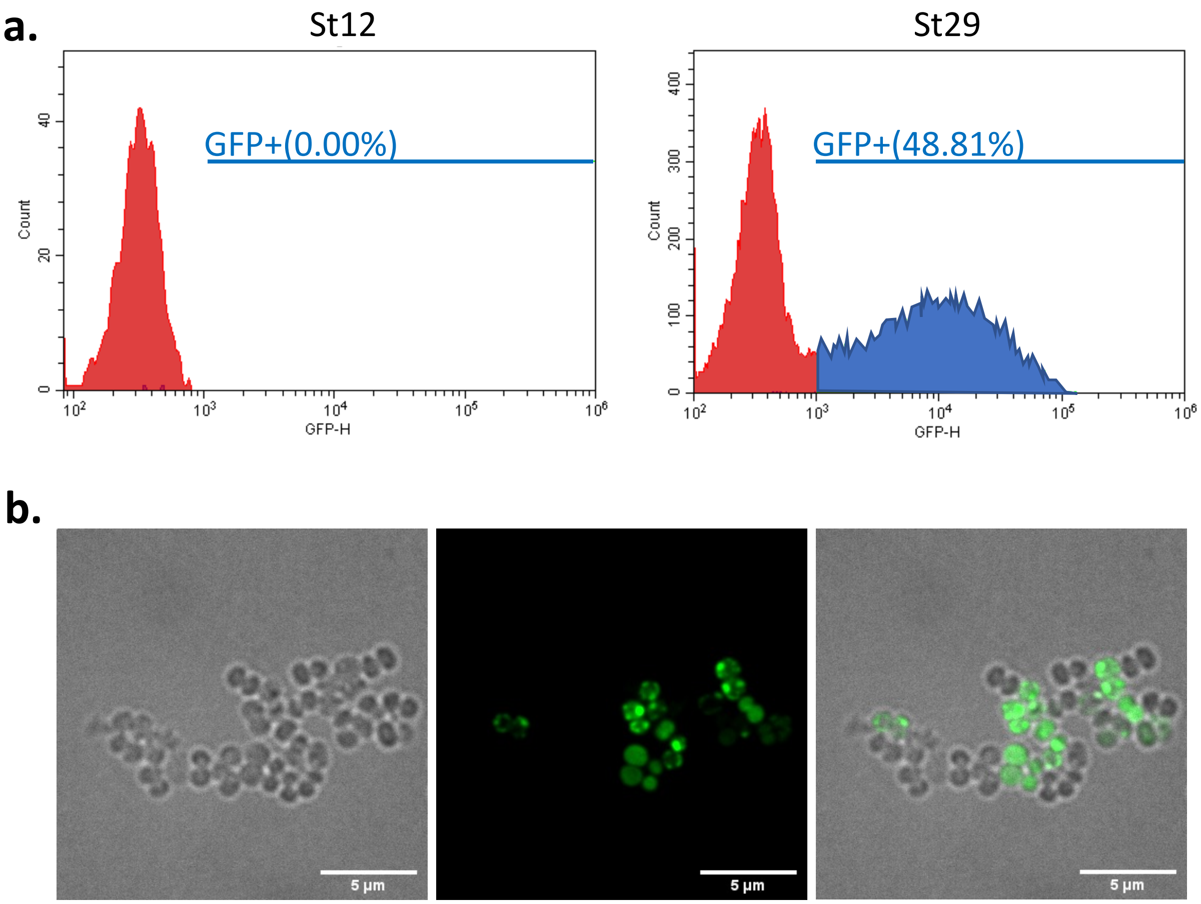

Supplementary Fig. 1. Flow cytometry and microscopy provide similar percentages of GFP-expressing competent cells (St29)

a. Classic flow cytometry experiment where the percentage of competent GFP-expressing cells is calculated by comparison of the fluorescence profile of a strain expressing GFP under the control of a competence-induced promoter (in this case, P_{comG}, St29) and a strain that does not express GFP (left, St12). The cells showing the maximum auto-fluorescence in the St12 culture, provide the threshold above which cells from the St29 culture are considered as competent (marked in blue).

b. Spinning-disk microscopy confirms that the percentage of competent GFP-expressing cells (St29, P_{comG}-*gfp*) is increased with our optimized protocol. In St29, the competent GFP-positive frequency reached 54.1 ± 12.7% (mean ± SD). The experiment has been repeated 5 times (biological replicates) with a least 500 cells counted in each experiment. Bar = 5 µm.

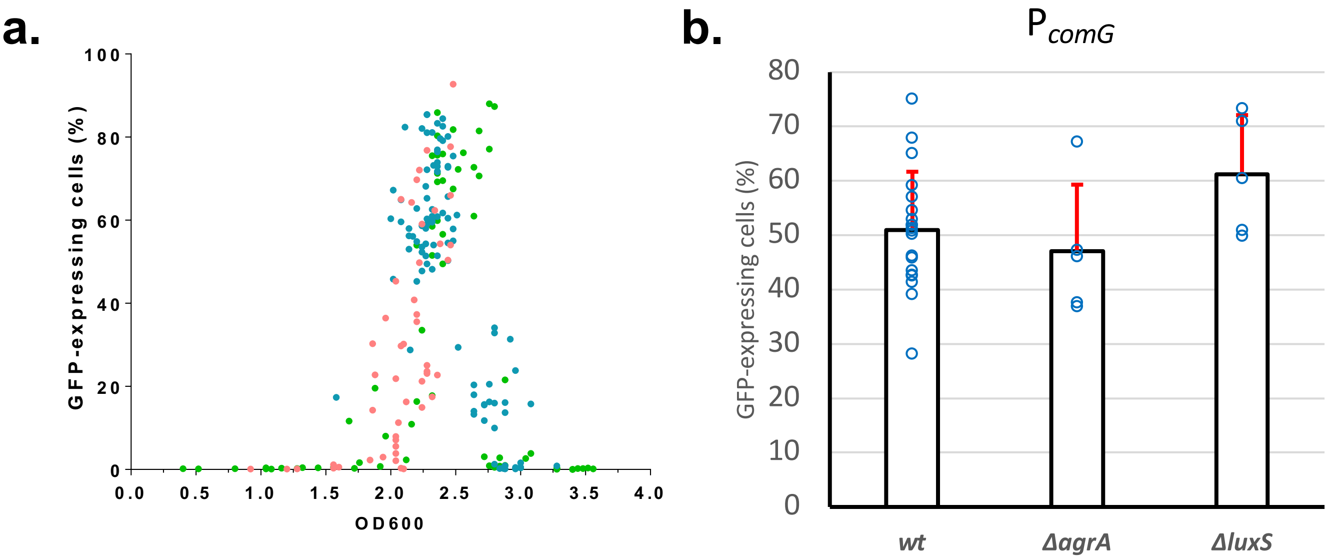

Supplementary Fig 2. Competence naturally develops at a specific cell density and growth phase in CS2.

a. Correlation between competence development (percentage of competent cells expressing the GFP under the control of the *comG* promoter, St29) and cell density (OD_{600}). This graph cumulates data obtained in 3 independent experiments (biological replicates). In each individual experiments, the results for all the diluted cultures (10^{-2} to 10^{-5}) are presented. Each experiment is materialized by a different color (green, blue or red dots). The competence window, during which the GFP expression was at its maxima, is allowed when ODs are between 2.2 and 2.6.

b. Percentage of competent cells expressing GFP under the control of the *comG* promoter in wild type (St29) or in the absence of *agrA* (St107) or *luxS* (St123) was determined after 22 hours of growth in CS2. Results are presented as mean ± SD. Each experiment has been repeated at least 5 times (biological replicates). Individual experiments are shown as blue circles.

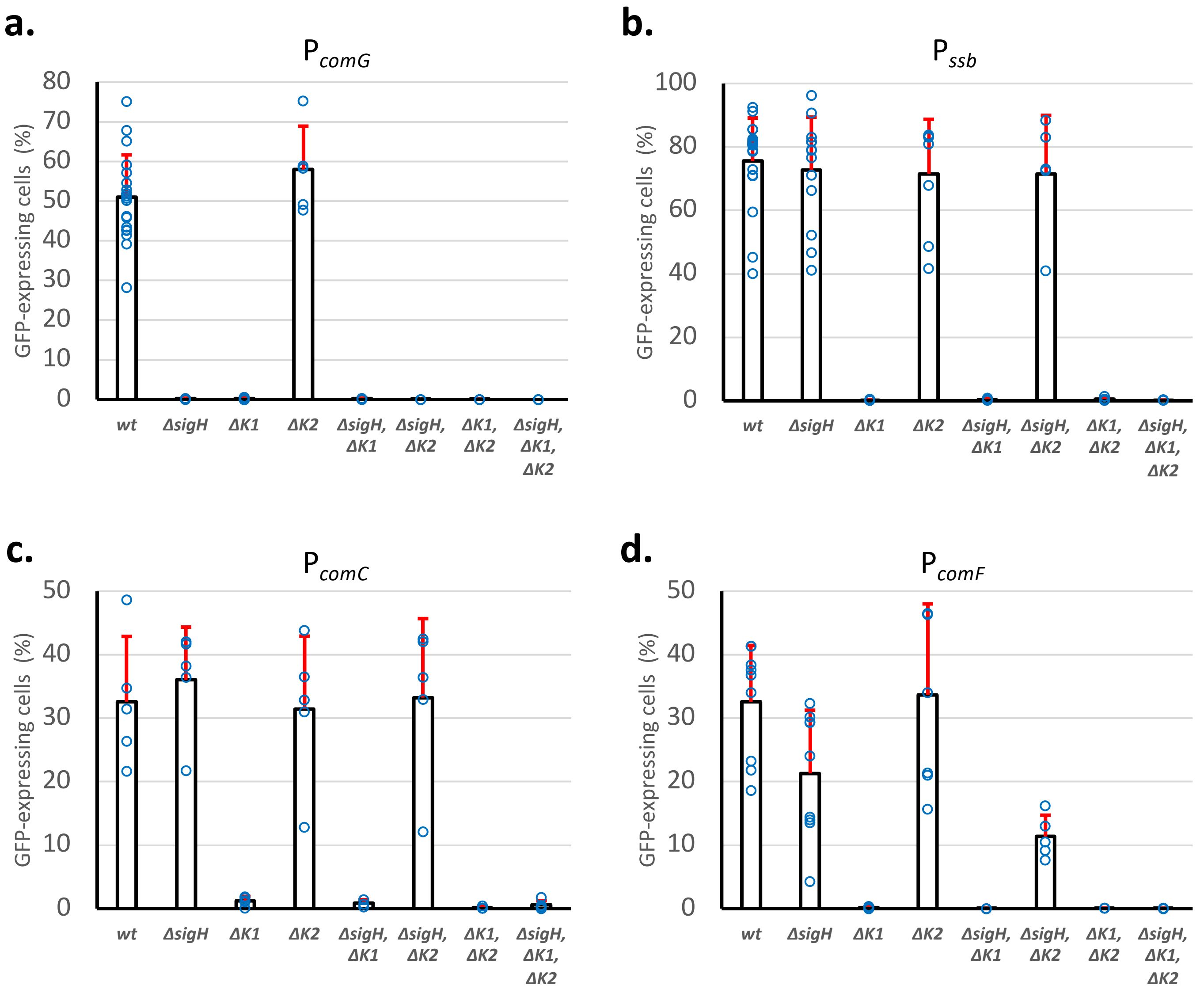

Supplementary Fig. 3. P_{comG}, P_{ssb}, P_{comC} and P_{comF} expression in all mutant strains

Percentage of competent GFP-expressing cells under the control of P_{comG} (St29, St51, St40, St 41, St47, St81, St77 and St69) (a), P_{ssb} (St50, St61, St64, St67, St75, St83, St79 and St71) (b), P_{comC} (St48, St60, St63, St66, St74, St82, St78 and St70) (c) and P_{comF} (St233, St235, St234, St236, St268, St269, St270 and St271) (d) in a wild type background or in the absence of *sigH*, *comK1*, *comK2*, *comK1/comK2*, *comK1/sigH*, *comK2/sigH or comK1,comK2/sigH* was determined after 21 hours of growth in CS2 medium. The results confirm those obtained in Fig. 2. Results are presented as mean ± SD. Each experiment has been repeated at least 5 times (biological replicates). Individual experiments are shown as blue circles.

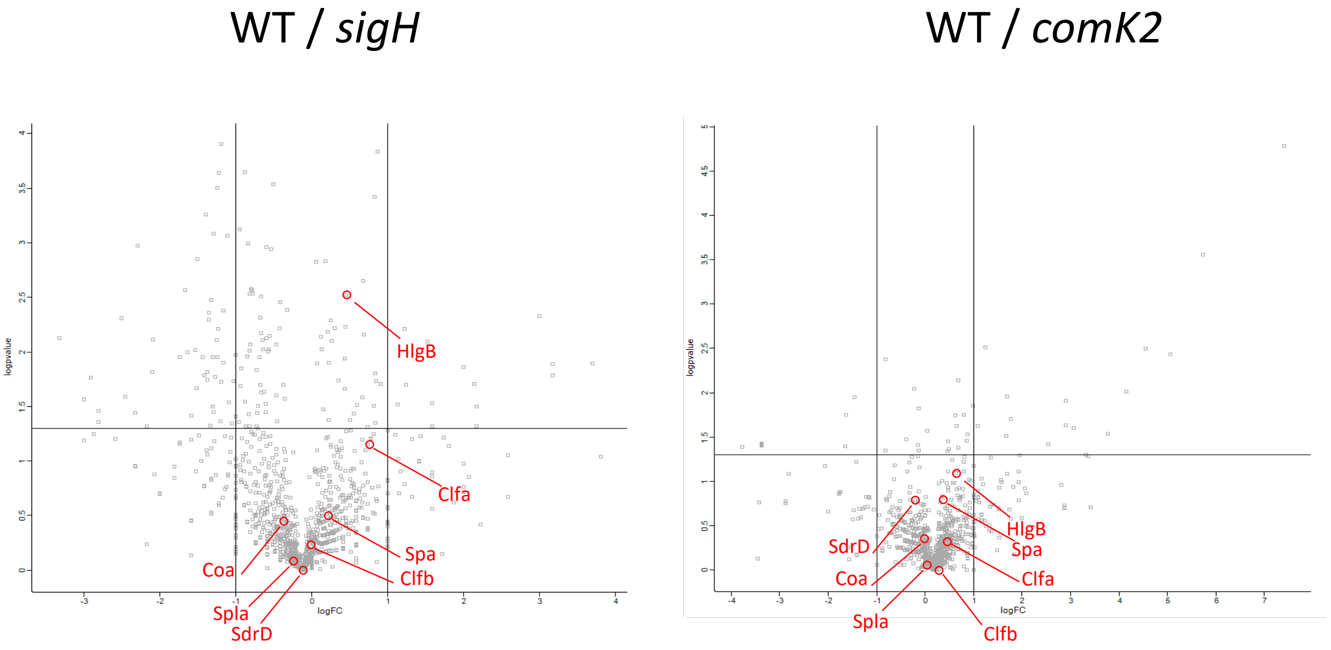

Supplementary Fig. 4. Exoproteome analysis shows no difference in the amount of virulence factors between wild type and *sigH* or *comK2* mutant strains.

Proteins are ranked in a volcano plot according to their statistical Pvalue (logpvalue) and their relative abundance ratio (log 2 fold change, logFC) between WT and *sigH* mutant (a) or WT and *comK2* mutant (b) strains. The horizontal line indicates the *p* value cut-off at 0.05. Only proteins that are found more (-1 < logFC) or less (logFC < 1) abundant than in the wt strain can be considered.

Few important virulence proteins were labeled and all of them were outside from the statistical constraints.

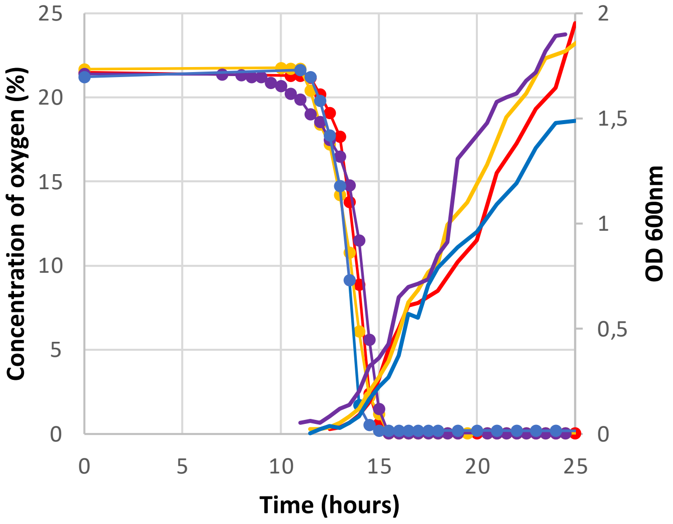

Supplementary Fig. 5. Growth in CS2 medium leads to microaerobic conditions.
To show reproducibility, growth (OD_{600nm}) and oxygen concentration were measured every 30 min. in four additional experiments (replicates of Fig. 5). Here, we confirm that as the OD increased, the concentration of oxygen quickly dropped. Once the oxygen concentration reached its minimum, the cultures paused and finally resumed growth.

Supplementary Tables

Locus tag N315	Gene name	Gene function	Δ*sigH*	Δ*comK1*	Δ*comK2*
SA1899	*ssb*	single-strand DNA-binding protein	0.506	0.000	0.494
SA1374	*comGA*	traffic ATPase	0.059	0.076	0.426
SA1373	*comGB*	polytopic membrane protein	0.043	0.057	0.435
SA1372	*comGC*	DNA transport machinery, major pilin	0.051	0.051	0.520
SA1371	*comGD*	DNA transport machinery, minor pilin	0.031	0.051	0.407
SA1370	*comGE*	DNA transport machinery, minor pilin	0.057	0.068	0.495
SA1369	*comGF*	DNA transport machinery, minor pilin	0.030	0.059	0.404
SA0705	*comFA*	ATP translocase	0.137	0.056	0.553
SA0706	*comFC*	amidophosphoribosyltransferases	0.101	0.043	0.536
SA1418	*comEA*	competence protein, membrane DNA receptor	0.185	0.253	0.424
SA1416	*comEC*	channel protein for DNA binding and uptake	0.530	0.660	0.795
SA1486	*comC*	leader peptidase (prepilin peptidase)	0.428	0.305	0.723
SA1128	*recA*	recombination protein	0.486	0.304	0.764
SA1092	*dprA*	dprA	0.306	0.426	0.454
SA1485	*radC*	DNA repair protein RadC	0.212	0.226	0.869
SA0858	*coiA*	competence protein CoiA	0.176	0.331	0.490

Supplementary Table 1. Genetic transformation genes expression is induced during natural and controlled by the competence central regulators.

Natural transformation genes differential expression between wild-type and *sigH*, *comK1* and *comK2* mutant strains. The ratios (normalized reads of mutant / wt) found in the last three columns reflect the importance of each central competence regulator in the regulation of a given gene. All the ratios below 0.5 (expression decreased by a 2-fold factor) are presented in red. Note that some genes present ratios between 0.4 and 0.5 in the *comK2* mutant strain. They do not appear in red as the effect associated to SigH and ComK1 is more important and because no effect could be observed with the GFP-reporter strains (see Supplementary Note 1).

In green, the *ssb* gene is the only one exclusively controlled by ComK1. The genes in blue (*comG* and *comF* operons) are controlled by both SigH and ComK1 (ratios < 0.1). The genes in yellow are controlled by SigH and ComK1 but the ratios are > 0.1.

Gene ID	Function	*Ratio*
SA2091	SarY, transcriptional activator	3,01
purD	Purine metabolism	3,01
SA0415	conserved hypothetical protein (ybjB)	3,02
feoB	major iron permease	3,02
SA1824	arsC Arsenate reductase (resistance to arsenic)	3,06
lytN	cell wall hydrolase	3,07
SA0632	Putative lipoprotein	3,12
SA2150	Putative hemin transport system permease protein HrtB	3,14
SA0136	similar to phosphonates transport permease	3,14
SA0408	hypothetical protein	3,17
clpB	Proteolysis in bacteria, stress	3,20
SA0704	hypothetical protein, DegV domain containing prot (operon with comF)	3,22
SA0530	alkaline phosphatase, aa (histidine) metabolism	3,24
SA2090	fnbB Fibronectin-binding protein, expresion affected by ClpP deletion	3,24
SA1878	epiA, lantibiotic epidermin precursor	3,24
purK	purine biosynthesis pathway	3,26
SA0333	Conserved hypothetical protein	3,30
SA1378	Conserved hypothetical protein	3,34
purF	purine biosynthesis pathway	3,37
SA1822	Toxin/antitoxin system?	3,37
SA0838	glyceraldehyde-3-phosphate dehydrogenase	3,41
SA0379	probable transposase	3,38
modA	Molybdate ABC transporter substrate-binding protein	3,46
purM	Purine metabolism	3,47
SA1978	similar to ferrichrome ABC transporter (permease)	3,49
SAS028	sRNA	3,51
SA1435	similar to acetyl-CoA carboxylase accB, fatty acid (lipid) metanolism	3,55
SA0283	serine protease?	3,59
SA0407	CHP (dipeptidyl aminopeptidases/acylaminoacyl-peptidases)	3,70
fmhC	secreted bacteriocin	3,72
SA1636	pathogenicity island saPI3	3,71
butA	Acetoin Reductase	3,77
radC	DNA repair protein	3,83
pyrP	uracil permease	3,90
SA0331	Efem/EfeO family lipoprotein	3,96
SA0837	Amino acid metabolism/transport	3,99
ndhF	NADH dehydrogenase subunit 5	4,14
purN	Purine metabolism	4,22
SA0412	hypothetical protein	4,28
SA0138	similar to alkylphosphonate ABC tranporter	4,30
SAS056	sRNA	4,34
SA0191	HlyD family secretion protein	4,42
SA0413	hypothetical protein (see SA0412, 0408, 0415)	4,48

Supplementary Table 2. SigH regulon during competence

List of genes, and their function, for which the expression is induced by more than a three fold factor by SigH. In the last column is provided the exact expression-induction factor associated to each gene under the control of SigH.

The table is in two parts (see next figure).

Gene ID	Function	*Ratio*
SA1152	hypothetical protein, hyothetical endotoxin? (see SA1151, 1153)	4,50
truncated-SA	Lytic regulatory protein truncated with Tn554	4,52
SA1418	comEA, DNA binding and uptake protein	4,53
SA1249	hypothetical protein, resistance to vancomycin?	4,87
purL	Purine metabolism	4,83
SA0858	Empbp, secretory extracellular matrix and plasma binding protein, fibrinogen-binding protein (see fnbB)	4,88
SA2338	ferrous iron transport protein B homolog (see SA1978)	5,51
SA1821	Toxin/antitoxin system? (see Mat&Met of https://europepmc.org/article/pmc/pmc6805077#free-full-text)	5,53
SA1151	hypothetical protein	5,74
lpl4	lipoprotein	5,74
SA0705	comFA; competence protein ComFA	5,77
uhpT	Sugar phosphate antiporter	5,95
purQ	Purine metabolism	6,04
SA2012	hypothetical protein	6,38
SA1153	DNA-dependent DNA polymerase family X	6,62
valS	valyl-tRNA synthetase, Protein Metabolism biosynthesis	6,65
SA0919	purS, phosphoribosylformylglycinamidine synthase, PurS protein	6,65
SAS040	Putative uncharacterized protein	6,96
folC	putative folylpolyglutamate synthase/dihydrofolate synthase	6,92
purC	Purine metabolism	7,08
SA0282	conserved hypothetical protein, Protein secretion system?	7,36
SA0706	comFC; competence protein ComFC	7,38
SA2197	putative DsbA homologue, protein disulfide bond formation	7,60
lpl7	lipoprotein	8,24
SA0576	toxin/antitoxin system?	11,69
SA2198	putative DsbA homologue, protein disulfide bond formation	11,80
trpB	tryptophan synthase subunit beta	12,44
SA1199	hypothetical protein, similar to anthranilate synthase component I	12,90
SA1370	ComGE, Late competence protein ComGE	13,49
SA1374	ComGA; competence protein ComGA	13,53
SA1372	comGC; competence protein ComGC	14,69
SA1373	comGB; competence protein ComGB	17,92
SA0575	toxin/antitoxin system? (see SA0576)	20,20
trpD	Anthranilate phosphoribosyltransferase	21,41
trpG	anthranilate synthase component II	22,18
trpC	Indole-3-glycerol phosphate synthase	22,52
SA1371	comGD; competence protein ComGD	23,53
trpF	N-(5'-phosphoribosyl)anthranilate isomerase	23,80
SA1369	ComGF, Late competence protein ComGF	24,56
SA2355	conserved hypothetical protein, contain Thioredox_DsbH and acetyl-transferase domains	25,23
SA1979	similar to toferrichrome ABC transporter	34,69

Supplementary Table 2 (end). SigH regulon during competence

List of genes, and their function, for which the expression is induced by more than a three fold factor by SigH. In the last column is provided the exact expression-induction factor associated to each gene under the control of SigH.

**Gene ID**	**Function**	***Ratio***
SA1833	annotated as a transcriptional activator (Str), Toxin/antitoxin system?	3,24
SA1486	ComC, Late competence protein ComC	3,24
groES	heat shock protein	3,27
grpE	response to hyperosmotic and heat shock by preventing the aggregation of stress-denatured proteins	3,28
SA1418	comEA, DNA binding and uptake protein	3,30
recA	Recombinase, catalyze the hydrolysis of ATP in the presence of single-stranded DNA	3,35
xprT	Xanthine phosphoribosyltransferase	3,48
truncated(radC)-2	DNA repair protein	3,59
SA1822	Toxin/antitoxin system?	3,78
SA1831	Toxin/antitoxin system?	3,82
SA2353	autolysins	4,16
SA1978	similar to ferrichrome ABC transporter	4,20
lacR	Repressor of the lactose catabolism operon	4,42
SA1709	ftnA, Ferritin-like protein	4,61
SA1820	operon with SA1821-1822, toxin/antitoxin?	4,80
hutH	Histidine ammonia-lyase	5,26
SA1821	Toxin/antitoxin system?	5,29
clpB	Proteolysis in bacteria, stress	6,64
folC	putative folylpolyglutamate synthase/dihydrofolate synthase	7,23
valS	valyl-tRNA synthetase	7,53
SA0704	conserved hypothetical protein, DegV domain containing prot	8,86
SA2198	Toxin/antitoxin system?	9,65
SA1635	hypothetical protein, Pathogenicity island SaPln3	10,28
SA2197	Toxin/antitoxin system?	10,16
SA1374	ComGA; competence protein ComGA	10,55
SA1370	ComGE, Late competence protein ComGE	11,22
SA1369	ComGF, Late competence protein ComGF	12,51
SA1373	comGB; competence protein ComGB	13,60
SA0705	comFA; competence protein ComFA	14,10
SA1372	comGC; competence protein ComGC	14,60
SA1371	comGD; competence protein ComGD	14,57
SA2355	conserved hypothetical protein, contain Thioredox_DsbH and acetyl-transferase domains	15,32
SA0706	comFC; competence protein ComFC	17,47
SA1979	similar toferrichrome ABC transporter	20,20
SA0575	Toxin/antitoxin system?	31,02
SA0576	Toxin/antitoxin system?	36,03
SA1898	hypothetical protein, similar to SceD precursor, able to cleave peptidoglycan and affects clumping and separation of bacterial cells	721,03
SA1899	ssb, Single-strand DNA-binding protein	2533,34

Supplementary Table 3. ComK1 regulon during competence

List of genes, and their function, for which the expression is induced by more than a three fold factor by ComK1. In the last column is provided the exact expression-induction factor associated to each gene under the control of ComK1.

Gene ID	Function	*Ratio*
SA1825	Toxin/antitoxin system?	3,17
SA0412	hypothetical protein	3,30
butA	Acetoin Reductase	3,42
SA2479	Polyisoprenoid-binding protein	3,47
SA1407	Conserved hypothetical protein, expression affected by ClpP deletion	3,51
SAS041	Hypothetical 62 aa protein	3,53
groES	heat shock protein, 10 kDa chaperonin, CtsR regulon (heat shock)	3,59
hutH	Histidine ammonia-lyase	3,63
groEL	heat shock protein, 60 kDa chaperonin, CtsR regulon (heat shock)	3,67
SA1824	Toxin/antitoxin system?	3,78
SA1978	similar to ferrichrome ABC transporter	3,88
SA1820	ribD, Riboflavin biosynthesis protein	3,89
dnaJ	Chaperone protein DnaJ	3,90
cysM	cysteine biosynthetic process from serine	3,91
pyrR	Regulates transcriptional attenuation of the pyrimidine nucleotide	4,00
SA1823	Arsenical pump membrane protein	4,18
SA0916	purE, phosphoribosylaminoimidazole carboxylase catalytic subunit	4,22
SA1821	Toxin/antitoxin system?	4,27
uhpT	Hexose phosphate transport protein	4,33
SA1822	Toxin/antitoxin system?	4,41
pyrC	Dihydroorotase	4,84
SA0481	hypothetical protein, CtsR regulon (heat shock)	4,91
purK	purine biosynthesis protein	5,09
purD	purine biosynthesis protein	5,10
purN	purine biosynthesis protein	5,29
clpC	CtsR regulon (heat shock)	5,30
SA0482	CtsR regulon (heat shock)	5,33
purH	purine biosynthesis protein	5,36
dnaK	Chaperone protein DnaK, CtsR regulon (heat shock)	5,41
purM	purine biosynthesis protein	5,60
ctsR	regulator of stress response, CtsR regulon (heat shock)	5,83
purF	purine biosynthesis protein	5,85
grpE	response to hyperosmotic and heat shock by preventing the aggregation of stress-denatured proteins, CtsR regulon (heat shock)	5,96
purC	purine biosynthesis protein	6,00
purL	purine biosynthesis protein	6,01
SA0919	Purine metabolism	6,11
purQ	purine biosynthesis protein	6,33
SA1596	aroK, Amino acid biosynthesis	6,50
hrcA	Heat-inducible transcriptional repressor, CtsR regulon (heat shock)	6,83
valS	valyl-tRNA synthetase, Protein biosynthesis	7,54
folC	putative folylpolyglutamate synthase/dihydrofolate synthase	8,07
trpA	tryptophan biosynthesis	8,45
SA1199	hypothetical protein	8,51

Supplementary Table 4. ComK2 regulon during competence

List of genes, and their function, for which the expression is induced by more than a three fold factor by ComK2. In the last column is provided the exact expression-induction factor associated to each gene under the control of ComK2.

The Table is in two parts (see next figure).

Gene ID	Function	*Ratio*
trpB	tryptophan biosynthesis	10,37
trpC	tryptophan biosynthesis	12,91
trpF	tryptophan biosynthesis	13,70
trpG	tryptophan biosynthesis	14,94
trpD	tryptophan biosynthesis	15,68
SA2355	conserved hypothetical protein, contain Thioredox_DsbH and acetyl-transferase domains	16,04
clpB	Chaperone protein, CtsR regulon (heat shock)	19,99
SA1979	similar toferrichrome ABC transporter	26,78
SA0576	Toxin/antitoxin system?	307,37
SA0575	Toxin/antitoxin system?	356,23

Supplementary Table 4 (end) ComK2 regulon during competence

List of genes, and their function, for which the expression is induced by more than a three fold factor by ComK2. In the last column is provided the exact expression-induction factor associated to each gene under the control of ComK2.

Gene ID	Function	*SigH*	*K1*	*K2*
capA	Capsular biosynthesis operon	**4,46**	2,14	**4,11**
capB	Capsular biosynthesis operon	**4,52**	-	2,71
capC	Capsular biosynthesis operon	**5,58**	-	**3,90**
capD	Capsular biosynthesis operon	**4,22**	-	**3,26**
capE	Capsular biosynthesis operon	**3,77**	-	**3,45**
capF	Capsular biosynthesis operon	**3,04**	-	2,58
capG	Capsular biosynthesis operon	2,84	-	2,52
capH	Capsular biosynthesis operon	2,41	-	-
capI	Capsular biosynthesis operon	2,32	-	2,09
capJ	Capsular biosynthesis operon	2,41	-	2,37
capK	Capsular biosynthesis operon	2,25	-	-
capL	Capsular biosynthesis operon	**3,04**	-	2,31
capN	Capsular biosynthesis operon	2,34	-	-
capO	Capsular biosynthesis operon	2,24	-	-
clfA	Clumping factor A	**6,39**	-	**3,31**
clfB	Clumping factor B	**4,21**	-	**3,46**
coa	Staphylocoagulase	-	-	2,49
fnb	Fibronectin-binding protein	2,70	**3,85**	-
fnbB	Fibronectin-binding protein	**5,64**	**8,02**	-
geh	Triacylglycerol lipase	**3,53**	-	-
hlgB	Toxin (Gamma-hemolysin component B)	-	2,12	-
hlgC	Toxin (Gamma-hemolysin component C)	2,59	2,24	**3,01**
icaA	Glycosyltransferase	**3,23**	**3,09**	-
icaB	Polysaccharide intercellular adhesin deacetylase	2,14	3,15	-
saeR	Transcriptional regulator, involved in the regulation of virulence factors	-	-	2,90
saeS	Histidine kinase, involved in the regulation of virulence factors	2,31	-	2,73
sdrC	Surface serine-aspartate repeat-containing protein C	2,24	-	-
sdrD	Surface serine-aspartate repeat-containing protein D	**4,15**	-	**3,58**
spa	Staphylococcal protein A (virulence factor)	**10,28**	2,11	**5,38**
splA	Serine protease	-	-	2,52
splB	Serine protease	-	-	2,48
splC	Serine protease	-	-	2,27
splD	Serine protease	-	-	2,39
splF	Serine protease	-	-	2,36
sspA	Serine protease	2,39	-	-
sspB	Serine protease	2,03	-	-
sspC	Serine protease	2,32	-	-

Supplementary Table 5. Expression of virulence-related genes is inhibited during competence. List of genes involved in virulence, and their function, that were found inhibited during the development of competence. The “inhibition” ratio calculated by comparison of the expression in wild type and mutant strains is presented in the last three columns. Ratios superior to 3 are in bold. ‘K1’ and ‘K2’ refer to ComK1 and ComK2.

Strains	Genotype/Construction	Source
**RN4220**	derivative of 8325-4, restriction minus, modification plus	41
**USA300**	MRSA clinical strain	42
**St012**	N315ex woφ	8
**St029**	N315ex woφ / pRIT-PcomG-gfp	8
**St048**	N315ex woφ / pRIT-PcomC-gfp	This study
**St050**	N315ex woφ / pRIT-Pssb-gfp	This study
**St233**	N315ex woφ / pRIT-PcomF-gfp	This study
**St037**	N315ex woφ ΔcomK1	This study
**St040**	ΔcomK1 / pRIT-PcomG-gfp	This study
**St063**	ΔcomK1 / pRIT-PcomC-gfp	This study
**St064**	ΔcomK1 / pRIT-Pssb-gfp	This study
**St234**	ΔcomK1 / pRIT-PcomF-gfp	This study
**St038**	N315ex woφ ΔcomK2	This study
**St041**	ΔcomK2 / pRIT-PcomG-gfp	This study
**St066**	ΔcomK2 / pRIT-PcomC-gfp	This study
**St067**	ΔcomK2 / pRIT-Pssb-gfp	This study
**St236**	ΔcomK2 / pRIT-PcomF-gfp	This study
**St045**	N315ex woφ ΔsigH	This study
**St051**	ΔsigH / pRIT-PcomG-gfp	This study
**St060**	ΔsigH / pRIT-PcomC-gfp	This study
**St061**	ΔsigH / pRIT-Pssb-gfp	This study
**St235**	ΔsigH / pRIT-PcomF-gfp	This study
**St044**	N315ex woφ ΔcomK1+ΔcomK2	This study
**St047**	ΔcomK1+ΔcomK2 / pRIT-PcomG-gfp	This study
**St074**	ΔcomK1+ΔcomK2 / pRIT-PcomC-gfp	This study
**St075**	ΔcomK1+ΔcomK2 / pRIT-Pssb-gfp	This study
**St268**	ΔcomK1+ΔcomK2 / pRIT-PcomF-GFP	This study
**St055**	N315ex woφ ΔcomK1+ΔsigH	This study
**St081**	ΔcomK1+ΔsigH / pRIT-PcomG-gfp	This study
**St082**	ΔcomK1+ΔsigH / pRIT-PcomC-gfp	This study
**St083**	ΔcomK1+ΔsigH / pRIT-Pssb-gfp	This study
**St269**	ΔcomK1+ΔsigH / pRIT-PcomF-GFP	This study
**St053**	N315ex woφ ΔcomK2+ΔsigH	This study
**St077**	ΔcomK2+ΔsigH / pRIT-PcomG-gfp	This study
**St078**	ΔcomK2+ΔsigH / pRIT-PcomC-gfp	This study
**St079**	ΔcomK2+ΔsigH / pRIT-Pssb-gfp	This study
**St270**	ΔcomK2+ΔsigH / pRIT-PcomF-GFP	This study
**St054**	N315ex woφ ΔcomK1+ΔcomK2+ΔsigH	This study
**St069**	ΔcomK1+ΔcomK2+ΔsigH / pRIT-PcomG-gfp	This study
**St070**	ΔcomK1+ΔcomK2+ΔsigH / pRIT-PcomC-gfp	This study
**St071**	ΔcomK1+ΔcomK2+ΔsigH / pRIT-Pssb-gfp	This study
**St271**	ΔcomK1+ΔcomK2+ΔsigH / pRIT-PcomF-GFP	This study
**St103**	N315ex woφ ΔagrA	This study
**St107**	ΔagrA / pRIT-PcomG-GFP	This study
**St122**	N315ex woφ ΔluxS	This study
**St123**	ΔluxS / pRIT-PcomG-GFP	This study
**St117**	N315ex woφ ΔsrrA	This study
**St145**	ΔsrrA / pRIT-PcomG-gfp	This study
**St147**	ΔsrrA / pRIT-Pssb-gfp	This study
**St118**	N315ex woφ ΔnreC	This study
**St158**	ΔnreC / pRIT-PcomG-gfp	This study
**St142**	N315ex woφ ΔairR	This study
**St177**	ΔairR / pRIT-PcomG-gfp	This study
**St250**	N315ex woφ ΔsrrA+ΔsigH	This study
**St197**	N315ex woφ pCN34	This study
**St252**	ΔsrrA+ΔsigH / pRIT-Pssb-gfp	This study
**NL10**	MRSA clinical isolate	16
**NL27**	MRSA clinical isolate	16
**NL36**	MSSA clinical isolate	16

Supplementary Table 6. Strains used in this study

Name	Nucleotide sequence	Descriptions
IM151	TAC ATG TCA AGA ATA AAC TGC CAA AGC	Used for verifying and sequencing the allelic replacement pIMAY constructs
IM152	AAT ACC TGT GAC GGA AGA TCA CTT CG	
KpnI-sigH KO-F-F	GCA GGT ACC GAC CCG CAT AAC TTG GGA TCA ATT TTA AGA	To amplify upstream of sigH used for the deletion of sigH
SalI-sigH KO-F-R	GCA GTC GAC CCC CTT CTA TCT AAA ATT TAA GGT TAG TTT AAT ATT GTT ACA TTC	
EcoRI-sigH KO-R-F	GCA GAA TTC AGC GCC TTA GGA CGT GAA TTG AAT TAT AAC GTG	To amplify downstream of sigH used for the deletion of sigH
XmaI-sigH KO-R-R	GCA CCC GGG CTG GTG TTT CTC GGC CAA ACA TAT CTA CTA ATA C	
sigH-OUT-F	TGC AGC GCT TAT TGC ACC ATA TGA ATA TGC	To verify the sigH deletion mutant
sigH-OUT-R	TTG CCC TCC CAC TCT TAA CAT TTC GTG A	
Kpn I-SA2017-up-F	GCA GGT ACC CAT ATG ACA CTC CCA ATG C	To amplify upstream of sa2107 used for the deletion of sa2017
Sal I-SA2017-up-R	GCA GTC GAC GTA GAA GTA CCT CCA AAA ATC AAT	
EcoRI-SA21017-down-F	GCA GAA TTC GCC CAT TAA CCT ATT TTT CAT A	To amplify downstream of sa2107 used for the deletion of sa2017
Sac I-SA2107-down-R	GCA GAG CTC ACA ATA TTT GAT GCC TGT GCT A	
check-mutant-SA2107-F	TGA AAG TCA GTC GTA CTC GAC AT	To verify the sa2107 deletion mutant
check-mutant-SA2107-R	GTT GCT CCC ATA TGC ATC TCA	
Kpn I-K1-up-F	GGT ACC GAA TGT TTA GTC ATG GTA CGT TGA TG	To amplify upstream of comK1 used for the deletion of comK1
Sal I-K1-up-R	GCA GTC GAC AAG CAA AAC CTC GCT TTA TTA AGT TT	
Sma I-K1-down-F	GCA CCC GGG TAT GCG GAA TGT TTT ATA	To amplify downstream of comK1 used for the deletion of comK1
Sac I-K1-down-R	GCA GAG CTC AAG GTT TGA TGT TAT CAG TGA ATG	
Kpn I-K2-up-F	GCA GGT ACC ATC TAT GCA TTG TTA CAT ATT CAT	To amplify upstream of comK2 used for the deletion of comK2
Sal I-RK2-up-	GCA GTC GAC ATA TAG TAC TCC TCG TAT AAT AAG	
EcoR I-K2-down-F	GCA GAA TTC GAA GCA CTT CAT TGA AAA TAC	To amplify downstream of comK2 used for the deletion of comK2
Sac I-K2-down-R	GCA GAG CTC AAG TGT CAG ATT CGT GTA AT	
check-mutant-comK1-F	TCT CAG GGA ATG CTA TGG TTA AAG T	To verify the comK1 deletion mutant
check-mutant-comK1-R	CAG TGG CTT ATT GGT CAC AGG	
check-mutant-comK2-F	GAG ACA ACA GCA AAT ATG ACA ACA AG	To verify the comK2 deletion mutant
check-mutant-comK2-R	GTG CAT GAA TAT TAC CAC TTC TAA TCG	
Kpn I-up-agrA-F	GCA GGTACC TTA GTG ACC ATG ATC ATA ATG TAT TTG AGT	To amplify upstream of agrA used for the deletion of agrA
Sal I-up-agrA-R	GCA GTCGAC TTC ACA TCC TTA TGG CTA GTT GTT AA	
EcoR I-down-agrA-F	GCA GAATTC AAT AAG ATA ATA AAG TCA GTT AAC GGC GTA	To amplify downstream of agrA used for the deletion of agrA
Sac I-down-agrA-R	GCA GAGCTC TCT GCT GAT ATG TTA TTT GAA CCA AG	
Check-mutant-agrA-F	GTA TTA CTT CTA TGG AAG TAG AGC CGT ATT	To verify the agrA deletion mutant
Check-mutant-agrA-R	GTG TAT CGC ACG AAT GAA GCA	
Kpn I-up srrA-F	GCA GGT ACC GAC ATT GAT TGC CAA AGG ACT AGT TGA G	To amplify upstream of srrA used for the deletion of srrA
EcoR I-up srrA-R	GCA GAA TTC TAC CTC CCA CAC ATG CTT TTC TTT ACA	
sma I-down srrA-F	GCA CCC GGG GGG CGT TGG GTA TAA ATT TGA GGT TAA ATC TA	To amplify downstream of srrA used for the deletion of srrA
Sac I-down srrA-R	GCA GAG CTC GCT CAT TAG TCA TAT CAC GAA CTG TCA C	
Check-mutant-srrA-F	GAA ATT ATC ACA AGC AGC AAT GGA AGT ACT	To verify the srrA deletion mutant
Check-mutant-srrA-R	TGG TAT TGT TAC AGA ACC GGA TGA AAT AAA AG	
Sal I-up nreC-F	GCA GTC GAC GAG CAT TCA ATC GAA AAG ATA GTT TTT GCT GAT G	To amplify upstream of nreC used for the deletion of nreC
Sma I-up nreC-R	GCA CCC GGG TCA AAT TGG AAT GTT CAA TGT AAC ATT GGT AC	
Not I-down nreC-F	GCA GCG GCC GC CGA GAG GAA ATG ATT ATC TTC TGG C	To amplify downstream of nreC used for the deletion of nreC
Sac I-down nreC-R	GCA GAG CTC GGTGACTGAATTTTGGCATACAC	
Check-mutant-nreC-F	GAT GAA TTA GGG TGT AAG TCA TGA TTA ATG AGG AC	To verify the nreC deletion mutant
Check-mutant-nreC-R	CTATTGAAGTTGCTACAACACTTCCAGCAC	
Sal I-up airR-F	GCA GTC GAC CAA GGT GCG TTA AAA TAT TTA ATT GAG GGC	To amplify upstream of airR used for the deletion of airR
EcoR I-up airR-R	GCA GAA TTC CAT GGG TTA TCT CCT TAA ATC AAG CTA TT	
sma I-down airR-F	GCA CCC GGG GTG TAA GTT TTA TCA AAT CTA GTG ATT GTG	To amplify downstream of airR used for the deletion of airR
Sac I-down airR-R	GCA GAG CTC GGT GAA ACA GCA TTC TAA TTG CAA TTA	
Check-mutant-airR-F	GGA GCG ATT TGA TGG AAC AAA GG	To verify the airR deletion mutant
Check-mutant-airR-R	GGC CAA TTT TAG TTG CAA TTT CTT CGC	
KpnI-up-luxS-F	GCA GGTACC AAT ATG TTG TCG TAG TTG TGC TAC	To amplify upstream of luxS used for the deletion of luxS
Sal I-up-luxS-R	GCA GTCGAC TTT GAA TTT CCT CCT ATT TAC TAC TCA A	
Sma I-down-luxS-F	GCA CCCGGG ATC TTA GTC AAT CAA GTT AAT CAG AAA AGC	To amplify downstream of luxS used for the deletion of luxS
Sac I-down-luxS-R	GCA GAGCTC AAA GCT GAA ACG CTT GAA GAA GC	
Check mutant-luxS-F	GCC ATC TAA AAT CAC ACC ATG CAC	To verify the luxS deletion mutant
Check mutant-luxS-R	CAT TAG GTT CTC AAA TGG TTG TAC TTG	

Supplementary Table 7. Primers used in this study

Name	Nucleotide sequence	Descriptions
**KpnI-up-comGA-F**	GCA GGTACC CGG AAG GTC CGA GTG TTA GTA AAT	To amplify upstream of comGA used for the deletion of comGA
**Sal I-up-comGA-R**	GCA GTCGAC CAC CTC CTA CAT ATA ATC ACG TAG GAG	
**Sma I-down-comGA-F**	GCA CCCGGG ACT ACA TTC TAA GAA GCG ACA ATT AAG TAA	To amplify downstream of comGA used for the deletion of comGA
**Sac I-down-comGA-R**	GCA GAGCTC CAT CTC TAT CAA TGT AAA CGC TTG AG	
**Check mutant-comGA-F**	CTA TTG TTG ATG TGG TTG TTA TTC CAG	To verify the comGA deletion mutant
**Check mutant-comGA-R**	CAA CCT GTT GAT TGT ATG TGA GCA G	
**KpnI-up-comC-F**	GCA GGTACC GGT ACT TAT CTA GAT ATT GCT AGG GAT	To amplify upstream of comC used for the deletion of comC
**Sal I-up-comC-R**	GCA GTCGAC ATG ACA ACC TCC TTA TGT AAA TTA TAG T	
**Sma I-down-comC-F**	GCA CCCGGG GTC ATT GCT GGT TTA GTT GCT TTA A	To amplify downstream of comC used for the deletion of comC
**Sac I-down-comC-R**	GCA GAGCTC TAC GAA GTC TGC TAA CAA CTC AA	
**Check mutant-comC-F**	CTA GTA TCG GTC TAG ACC ATA CAG ATA	To verify the comC deletion mutant
**Check mutant-comC-R**	TTT GAT GCT GAA GGA TAT CTC AAC C	
**KpnI-up-ssb-F**	GCA GGTACC TAA TGC CAG GCG TAT TAA TTA CTG	To amplify upstream of ssb used for the deletion of ssb
**Sal I-up-ssb-R**	GCA GTCGAC GTA GTG TGT GAT TCA CCT CCT ATG	
**Sma I-down-ssb-F**	GCA CCCGGG ATC CAA TTA TCC TAA ACA TCC TTA ATA TAC	To amplify downstream of ssb used for the deletion of ssb
**Sac I-down-ssb-R**	GCA GAGCTC ACT ATT GAT TAG CTA TGC ATA AAT GGC	
**Check mutant-ssb-F**	AGT ATC AGG AAA CGA ACC ATT CTT TCA A	To verify the ssb deletion mutant
**Check mutant-ssb-R**	GGA ACA TAT CGA GAA TTC CCC GAT ATA T	
**Gibson-pRIT-gfp-F**	GGA TCC GGG AGG CCG TTT	To amplify the linear pRIT used for the reporter plasmid construction by Gibson
**Gibson-pRIT-gfp-R**	CTC TCA GTA CAA TCT GCT CTG ATG CCG	
**pRIT-PcomF-F**	ATA TGT GTT CGT GAA TTC CAG TTT GAT CAT AAT TCA GTG TTA CTA TAC ATG GTA CTG	To amplify the promoter of comF used for the reporter plasmid constructoin by Gibson
**gfp-PcomF-R**	AAA CGG CCT CCC GGA TCC TGT TAT TTT ATA TCT TGT TAC ATT ATC CAT TCG ACC CAG TGA TAT	
**pRIT-Pssb-F**	CGG CAT CAG AGC AGA TTG TAC TGA GAG TGC GGT AAT GCC AGG CGT ATT AAT TAC TG	To amplify the promoter of ssb used for the reporter plasmid construction by Gibson
**gfp-Pssb-R**	AAA CGG CCT CCC GGA TCC TCT CCC GAC AAT TAC GAT TTT ATT TAG CAT GTA GTG TG	
**pRIT-PcomC-F**	CGG CAT CAG AGC AGA TTG TAC TGA GAG AAG CCT AAC GTT CCA GTG ATA TAT GCT GTT AAA AAT G	To amplify the promoter of comC used for the reporter plasmid construction by Gibson
**gfp-PcomC-R**	AAA CGG CCT CCC GGA TCC TGC AGC TAT AAG ATA ACA ATA CTA CCA AAT GAC AAC CT	
**pRIT-PcomK1-F**	CGG CAT CAG AGC AGA TTG TAC TGA GAG GTA ACG ACA AAT ATA GAA CTT GGG CAT GGA	To amplify the promoter of comK1 used for the reporter plasmid constructoin by Gibson
**gfp-PcomK1-R**	AAA CGG CCT CCC GGA TCC AAG CAA AAC CTC GCT TTA TTA AGT TTT AAA CC	
**pRIT-PcomK2-F**	CGG CAT CAG AGC AGA TTG TAC TGA GAG GAC CAT CAC GAT ACA TCA TTT CAT TAG TGA	To amplify the promoter of comK2 used for the reporter plasmid construction by Gibson
**gfp-PcomK2-R**	AAA CGG CCT CCC GGA TCC ATA TAG TAC TCC TCG TAT AAT AAG TTG TTA ATT TAA TGG	
**pRIT-PsigH-F**	CGG CAT CAG AGC AGA TTG TAC TGA GAG AAC AGG TGC AAT TGA ACA TGT ACC AGT TA	To amplify the promoter of sigH used for the reporter plasmid construction by Gibson
**gfp-PsigH-R**	AAA CGG CCT CCC GGA TCC GTA TTA AAC TAA CCC CTT CTA TCT AAA ATT TAA GGT TAG	
**pRIT-X-F**	GGC TTA ACT ATG CGG CAT CAG AGC	Used for verifying and sequencing the pRIT constructs
**pRIT-X-R**	GAA TTG TGA GCG GAT AAC AAT TTC ACA CAG G	

Supplementary Table 7 (end). Primers used in this study

Supplementary Note

Supplementary Note 1. Comparison of the results obtained in the RNA-sequencing experiments and the GFP-reporter strains.

Overall, we could define three classes of genetic transformation genes: (i) Class I: genes for which SigH and ComK1 are both essential, (ii) Class II: genes (ssb) exclusively controlled by ComK1 (and (iii) Class III genes are controlled by SigH and ComK1 even though their impact is not as important.

However, compared to the GFP reporter strains (**Fig 2a-d**), the impact of the regulators deletion does not always provide the same result by RNA-sequencing. Indeed, the results are confirmed for the *comG or ssb* expression, controlled either by SigH and ComK1 or only by ComK1. However, if we consider the *comF* or *comC* expression, ComK1 and SigH seem to have a small impact through RNA-sequencing **(Supplementary Table 1)**, while only ComK1 appeared essential using GFP-reporter strains (**Fig 3C**).

Importantly, the differences observed between GFP and RNA-sequencing experiments could be explained by the fact that GFP accumulates during time (because of its stability) while mRNA contents evolve rapidly (because of a high turnover [29]). Therefore, samples of GFP-reporter strains could be taken later into stationary phase, while we extracted the mRNA content at a specific time during the transition to stationary phase. Interestingly, these results suggest that all the genetic transformation genes are not expressed exactly at the same time and that specific regulations (involving one or several regulators) could be at play.
