# Supplementary material for: The complex regulation of competence in Staphylococcus aureus under microaerobic conditions
Source: Commun Biol. 2023 May 12;6:512. doi: 10.1038/s42003-023-04892-1 (PMC10182052; doi:10.1038/s42003-023-04892-1)
Supplement: Supplementary file 3 — Description of Additional Supplementary Files [file 42003_2023_4892_MOESM3_ESM.pdf]

Description of Additional Supplementary Files

File name: Supplementary Data

Description: Source Data for this paper
