# Supplementary material for: The complex regulation of competence in Staphylococcus aureus under microaerobic conditions
Source: Commun Biol. 2023 May 12;6:512. doi: 10.1038/s42003-023-04892-1 (PMC10182052; doi:10.1038/s42003-023-04892-1)
Supplement: Supplementary file 1 — Mirouze_Peer review file [file 42003_2023_4892_MOESM1_ESM.pdf]

nature portfolio

Peer Review File

The complex regulation of competence in Staphylococcus aureus under Microaerobic conditions

Open Access This file is licensed under a Creative Commons Attribution 4.0 International License, which permits use, sharing, adaptation, distribution and reproduction in any medium or format, as long as you give appropriate credit to the original author(s) and the source, provide a link to the Creative Commons license, and indicate if changes were made. In the cases where the authors are anonymous, such as is the case for the reports of anonymous peer reviewers, author attribution should be to 'Anonymous Referee' followed by a clear attribution to the source work. The images or other third party material in this file are included in the article's Creative Commons license, unless indicated otherwise in a credit line to the material. If material is not included in the article's Creative Commons license and your intended use is not permitted by statutory regulation or exceeds the permitted use, you will need to obtain permission directly from the copyright holder. To view a copy of this license, visit http://creativecommons.org/licenses/by/4.0/.

Reviewers' comments:

Reviewer #1 (Remarks to the Author):

The development of natural competence by S. aureus has been a big mystery. Recently, however, several manuscripts suggest it may be an important process for this bacterium. The current manuscript analyses this phenomenon and provides interesting results identifying new players in this this process. Here are some comments and suggestions that I hope the authors find of interest:

- Lane 105 (and other places in the manuscript): since the reporter gene was cloned under the control of the comG promoter, the authors assume that the cells expressing the gfp reporter are competent. The low transformation efficiencies observed does not support that affirmation. What is the evidence showing that higher expressions of the reporter genes correlate with an increased ability of the cells to uptake extracellular DNA? This is an important question because this assumption is used constantly through the manuscript.

- Can the same results be obtained using different S. aureus strains?

- I'm not sure the term natural competence is appropriate here. The authors must use a completely artificial media to induce "competence".

- Since the authors propose that comK2 is not involved in competence (Fig. 1c), it is unclear to me why the results obtained with the mutant in this gene are further included in all the analyses (line 180).

- Related to the previous comment, it would be nice to highlight what genes are controlled by both SigH and ComK1. One could imagine that these genes may be important to develop competence. Moreover, this hypothesis could be tested by analyzing the impact of their mutation in the efficiency of transformation. I think 2-3 mutants should be sufficient to prove the hypothesis.

- The authors suggest that competence limits virulence. In addition to the expression of the virulence gene, it would be interesting to demonstrate that the final amount of the virulence factor that is produced is reduced when the cells enter in competence.

Reviewer #2 (Remarks to the Author):

Emergence of antibiotic multi-resistant strains of Staphylococcus aureus is a dramatic health problem worldwide. Understanding transfer mechanisms of antibiotic resistance genes in this key pathogen is of major importance. In 2012, a first report showed that S. aureus was capable to activate competence for DNA transformation at a very low level but sufficient to allow the acquisition of large pieces of DNA (including antibiotic resistance genes). The work by Feng et al. reports a significant improvement (more than 100-fold) of S. aureus transformation rate with planktonic cells. Moreover, the authors showed that a large fraction of the cell population (>60%) activates this developmental process. Those novel conditions allowed the investigation of the functional roles of the 3 central regulators of competence (i.e. SigH, ComK1, ComK2) in S. aureus. The trancriptomics analyses showed the key role played by SigH and ComK1 in the direct control of genes needed for transformation. Peripherical functions related to the whole program of competence development were also highlighted as well as the repression a large set of virulence genes. The ComK2 regulon does not include strict competence genes but overlap with the SigH-ComK1 regulons. Finally, the authors showed that that microaerobic conditions are key to activate competence in S. aureus and that the SrrAB two-component system sensing oxygen availability is potentially involved in the control of SigH.

Although two recent reports in Nature Communications (spring 2022) shed new light on competence development in S. aureus, the competence conditions identified in this work will be easy to reproduce as they do not need damaging stresses or growth in biofilms. This opens the door to a better understanding of the complexity of competence regulation in this species, as well as facilitating genetic modifications in the future. Those novel conditions already allowed to better identify the regulons of the central competence regulators in a more relevant way (i.e. through gene deletion) that the previous report based on their overproduction (that could lead to artefactual results). Moreover, oxygen limitation was identified as a key parameter for activation, which make sense regarding the lifestyle of S. aureus during infection. Globally, the manuscript is logically organized and convincing data are provided. Nevertheless, I have a list of suggestions to improve the global presentation and to better highlight the reproducibility of the results.

Comments on formal presentation:

1. The current version of the summary is not really informative regarding the key results obtained during this work and could be improved.

2. The transcriptomic work and the analysis of SigH, ComK1, and ComK2 regulons is summarized in Table 1 in the main text. This table is not very informative with vague indications (+, +++) and the most important data are presented as supplemental figures. As a large part of manuscript describes those regulons, I would suggest to replace Table 1 by a composite figure that could include Supp Fig. 6 and Supp Fig 10 to show the overlap between the 3 regulons as top panels. For the five functions reported in Table 1, you can generate overlapping circles for the 3 regulons per function for up and down-regulated genes (two bottom panels).

3. The reproducibility of data is not fully reported. Indications of technical or biological replicates are missing in most cases. Do the error bars correspond to SEM/SD or confidence intervals? In bar charts, please show individual values as dots. For microscopy, how many cells per replicate were examined? Please indicate that you show a mean value. I would suggest to include all information regarding reproducibility in the figure legends. I would also suggest a homogenous presentation through the whole set of figure legends (including Supp material)

-Fig 1a and b: one representative experiment? How many biological replicates? Between 18 and 24h in the legend, but shown between 16 and 22h in the fig panel. Fig. 1c: 10 replicates, biological or technical? Numbers are mean values +/- SD or SEM?
-Associated Supp Fig 1: those data are exactly the same as presented in fig 1a and 1b. I do not see the interest to show them again in a supplementary figure.
-Associated Supp Fig 2b: how many replicates? How many cells were examined per replicate?
-Fig. 2: mean values +/- SD? Show individual values. 5 replicates but technical or biological?
- Associated Supp Fig 3a: data are represented as mean +/- SEM, I do not see mean and error bars?
- Associated Supp Fig 3b and Supp Fig 4, Fig. 3: Numbers are mean values +/- SD or SEM? Show individual values. Each experiment in each condition has been repeated at least 5 times – this statement is not very clear.
-Fig 4: one representative experiment? How many repetitions?

4. Tables are presented as figures: Fig. 1c, Supp Fig. 5, Supp Fig. 7, Supp Fig. 8, and Supp Fig 9 and Supp Fig 11. For Fig 1c, I would suggest to present the data as a bar chart with values on the top or extract it as a separate table. For the others, I would name them tables. Please note that for Supp Fig/Table 5, values (ratio?) and color cut-off are not described.

5. Please provide a GEO number for the whole set of RNAseq data

Scientific comments:

6. L113-119: DNA transformation with plasmids and chromosomal DNA. I was wondering if the authors have performed trials with linear PCR products including an antibiotic resistance marker and recombination arms (>1 kb). Generally, such donor DNA can lead to high transformation rates. If done, it could be interesting to include those results.

7. L180-L215: contribution of ComK2 to the whole program of competence development. The authors should better explain why ComK2, that do not control any essential gene required for DNA transformation, is involved in the whole program. They should better explain the overlapping of regulons based on the figure I suggest to replace Table 1. L188-189, please give references.

8. L232: SrrAB might control the sigH gene. It should be interesting to show if the promoter of sigH is directly controlled by SrrAB using a PsigH-gfp fusion.

9. L249-250 and L285-286: growth in CS2 medium leads to oxygen limitation. Could you give an explanation here or in the discussion (L285-286)? Oxygen limitation probably result in a dramatic change in carbon metabolism which could be more compatible with competence development. A couple of sentences in the discussion should report this and refer to the published work of Cordero et al. (ref 35).

10. L281: transformation protocol, dilutions were incubated with shaking. Could you explain why cultures were agitated, knowing that microaerobic conditions are required for optimal competence activation. Is it for GFP maturation? L465, why agitation during the transformation assay?

Minor comments/corrections:

11. Page numbers are missing.
12. In all numerical values, replace the comma by a dot in figures and text (e.g. L115, L117, …)
13. Check carefully gene and protein names for capitals and italics (e.g. L147, PcomF; …). For promoters, the gene name in italics is underscored.
14. The use of “would” is not always appropriate. Please check

<u>ANSWER TO THE REVIEWERS</u>

Microaerobic conditions and the complex regulation of natural competence in *Staphylococcus aureus*

Shi Yuan Feng [‡,1], Yolande Hauck [‡,1], Fedy Morgene [1], Roza Mohammedi [1] and Nicolas Mirouze [*,1]

[1] Université Paris-Saclay, CEA, CNRS, Institute for Integrative Biology of the Cell (I2BC), 91198, Gif-sur-Yvette, France

[‡] Co-first authors

[*] Corresponding author

Email: nicolas.mirouze@i2bc.paris-saclay.fr

<u>Referee expertise:</u>

Referee #1: Mechanisms of horizontal gene transfer

Referee #2: Bacterial transformation and competence

Reviewer #1 (Remarks to the Author):

The development of natural competence by S. aureus has been a big mystery. Recently, however, several manuscripts suggest it may be an important process for this bacterium. The current manuscript analyses this phenomenon and provides interesting results identifying new players in this this process. Here are some comments and suggestions that I hope the authors find of interest:

We would like to thank Reviewer #1 for his/her time to review our manuscript and for the helpful comments and suggestions made. Our answers are presented below and the changes or precisions suggested by Reviewer #1 clearly improved our manuscript.

- Lane 105 (and other places in the manuscript): since the reporter gene was cloned under the control of the comG promoter, the authors assume that the cells expressing the gfp reporter are competent. The low transformation efficiencies observed does not support that affirmation. What is the evidence showing that higher expressions of the reporter genes correlate with an increased ability of the cells to uptake extracellular DNA? This is an important question because this assumption is used constantly through the manuscript.
We agree with Reviewer #1 that the question of the correlation between *comG* expression, competence induction and transformation efficiencies is important.

Importantly, in the present study we used the strain published by Morikawa and colleagues (Morikawa *et al*., Plos Pathogens, 2012) in order to be able to compare our results to theirs (St29: N315ex woϕ, pRIT-P_{comG}-*gfp*). In the present manuscript we assumed that cells expressing the *gfp* reporter under the control of the *comG* promoter are competent as this correlation has been extensively shown in the literature. Based on the same assumption, Morikawa already used this strain (expressing the *gfp* under the control of the *comG* promoter) to evaluate the percentage of cells inducing competence through microscopy in his 2012 paper.

Here is a short paragraph to fully explain our reasoning. In Morikawa *et al*., 2012, the authors presented the first protocol leading to genetic competence development. Under these conditions, 1.6% of a wild type strain culture would induce competence (percentage evaluated by the detection of cells expressing the *gfp* under the control of the *comG* promoter), leading to barely detectable transformation efficiencies around 10^{-10}.
In the present manuscript, we show an optimized protocol that improves the percentage of cells inducing competence (and therefore expressing the *comG* operon) in a wild-type background (around 50%) and the transformation efficiency to 4. 10^{-6}.
We therefore assumed that the difference between Morikawa's results (1.6% of competent cells) and ours (close to 50%) explained the difference between our transformation efficiencies (10^{-10} vs 4. 10^{-6}).

Finally, we agree that the transformation efficiencies that we measure with a *S. aureus* wild type strain are not as high as with other model organisms (*S. pneumoniae* or *B. subtilis*), even though some new strains tested improved our results (see next comment). However, we strongly believe that generating hundreds of transformants in 100ul of culture represents a substantial achievement to prove that natural transformation is important for *S. aureus* genomic plasticity but also a demonstration of the practicality of this mode of horizontal gene transfer as a powerful genetic tool in *S. aureus*.

- Can the same results be obtained using different S. aureus strains?
We agree with Reviewer #1 that this is an important question. We optimized the protocol using the N315 S. *aureus* strain. One could therefore ask if this protocol also "works" with other laboratory or clinical strains. Interestingly, when we grew, following our optimized protocol, two laboratory strains (RN4220 and USA300) and three clinical isolates (NL10, 11 et 33), we obtained diverse but interesting results.
First, RN4220 displays a very high transformation efficiency reaching 10^{-4} (higher than N315). This could be explained by the fact that RN4220 lacks restriction/modification systems that could limit natural transformation events in N315. On the other side, strain USA300 shows a transformation efficiency lower than N315. This could mean that competence regulation might be different in USA300 (regarding the timing for example) and that further investigation will be required to find conditions leading to the optimized natural transformation of USA300.
Finally, we were delighted to observe that our protocol also leads to clinical isolates natural transformation. Even though the transformation efficiencies are lower than that of N315, this result shows that our protocol is robust and can be transposed to many different *S. aureus* strains and could be used in the future as a powerful genetic tool.

These results are presented in the new Figure 1d and line 124

- I'm not sure the term natural competence is appropriate here. The authors must use a completely artificial media to induce "competence".
We agree with reviewer #1. We changed to "natural" transformation throughout the manuscript.

- Since the authors propose that comK2 is not involved in competence (Fig. 1c), it is unclear to me why the results obtained with the mutant in this gene are further included in all the analyses (line 180).
In Fig. 1c, we show that ComK2 is not involved in the control of natural transformation. However, in the paragraph starting line 180, we demonstrate that despite the fact that ComK2 does not control natural transformation, it is still required for a full competence development by activating "peripherical" functions such as the general stress response, toxin-antitoxin systems and amino and nucleic acid metabolism. These peripheral functions have been described in the past in other model organisms, and we think it is important to confirm that it is also the case in *S. aureus*.

In addition, we have preliminary data suggesting that ComK2 could play other roles in experimental conditions that are different than those presented here. Therefore, in order to establish a clear and complete reference for this future study, we feel that we needed to include ComK2 in all our analyses.

- Related to the previous comment, it would be nice to highlight what genes are controlled by both SigH and ComK1. One could imagine that these genes may be important to develop competence. Moreover, this hypothesis could be tested by analyzing the impact of their mutation in the efficiency of transformation. I think 2-3 mutants should be sufficient to prove the hypothesis.
The first part of Reviewer #1' comment is very similar to the 2nd comment of Reviewer #2. See below for details as well as the new Fig. 3.

Then, Reviewer #1 proposes that some of the genes controlled by both SigH and ComK1 could be involved in competence development.
To summarize what has been shown in other model organisms, we can say that in response to environmental signals (usually stresses), bacteria induce regulatory cascades ultimately leading to the expression of the competence central regulators. All these regulatory steps are performed by the proteins encoded by the so-called "early" competence genes. Once activated, these central competence regulators promote the expression of the "late" competence genes among which are found the genes responsible for natural transformation.
Importantly, our RNA-sequencing results are based on comparison of the RNA content between a wt strain and strains in which the gene encoding the central competence regulators is deleted. This is how we can present the regulons (i.e. late competence genes) controlled by each regulator. Consequently, activation of the late competence genes, controlled by SigH and ComK1, is thought to be the final output of competence development. Therefore, we believe that there is a very low probability that late competence genes could be involved in the early regulatory steps leading to the development of competence. To our knowledge, the only example of late competence genes involved in the regulation of competence is DprA in *S. pneumoniae*. In this case, DprA controls the shut off of competence and not its activation.

- The authors suggest that competence limits virulence. In addition to the expression of the virulence gene, it would be interesting to demonstrate that the final amount of the virulence factor that is produced is reduced when the cells enter in competence.
We agree with reviewer #1 that it would be interesting to verify that the final amount of virulence factors is reduced during competence development in wild type cells in comparison to central competence regulators mutants. This is why we conducted a quantitative proteomic study to compare the exoproteome of a wild type strain to that of strains deleted for the genes encoding SigH and ComK2, the two main inhibitors of virulence genes expression.

Unfortunately, this comparative proteomic study led us to conclude that there is no significant difference in the amount of virulence factors secreted by wild type and *sigH* or *comK2* mutant S. aureus cells at maximum competence.

In order to explain this result, we can say that:

- Competence development is bistable, with two populations co-existing, competent and non-competent cells each representing 50% of the total population. This means that the production of virulence factors by non-competent cells would dilute the difference observe between wt and mutant competent cells.
- In addition, the number of virulence genes transcripts detected in wild type and mutant strains was quite low (even though significantly higher in the absence of *sigH* or *comK2*). If virulence factors have been massively produced and accumulated early during the culture, as the concentration of oxygen was decreasing and before competence development, the expression inhibition detected at a specific time point would not lead to an important difference.

Even though no difference could be observed in the total amount, we still believe that inhibition of the virulence genes expression is an important result. Indeed, this probably means that the development of competence, which is very energy consuming, requires to shut down other "cellular functions" (as virulence factors production) in order to concentrate on natural transformation. Inhibition of such energy consuming cellular processes (i.e. replication,

division, cell wall synthesis…) during competence development has already been described in other model organisms.

We present these proteomic data in the new Supplementary Fig. 5, discuss these results line 221 and provide experimental information as well as a deposit number in material and methods. Raw mass spectrometry proteomics data can be seen by the reviewers:
Reviewer account details:
Username: reviewer_pxd040550@ebi.ac.uk
Password: HPMHiKTA

Reviewer #2 (Remarks to the Author):

Emergence of antibiotic multi-resistant strains of Staphylococcus aureus is a dramatic health problem worldwide. Understanding transfer mechanisms of antibiotic resistance genes in this key pathogen is of major importance. In 2012, a first report showed that S. aureus was capable to activate competence for DNA transformation at a very low level but sufficient to allow the acquisition of large pieces of DNA (including antibiotic resistance genes). The work by Feng et al. reports a significant improvement (more than 100-fold) of S. aureus transformation rate with planktonic cells. Moreover, the authors showed that a large fraction of the cell population (>60%) activates this developmental process. Those novel conditions allowed the investigation of the functional roles of the 3 central regulators of competence (i.e. SigH, ComK1, ComK2) in S. aureus. The trancriptomics analyses showed the key role played by SigH and ComK1 in the direct control of genes needed for transformation.

Peripherical functions related to the whole program of competence development were also highlighted as well as the repression a large set of virulence genes. The ComK2 regulon does not include strict competence genes but overlap with the SigH-ComK1 regulons. Finally, the authors showed that that microaerobic conditions are key to activate competence in S. aureus and that the SrrAB two-component system sensing oxygen availability is potentially involved in the control of SigH.

Although two recent reports in Nature Communications (spring 2022) shed new light on competence development in S. aureus, the competence conditions identified in this work will be easy to reproduce as they do not need damaging stresses or growth in biofilms. This opens the door to a better understanding of the complexity of competence regulation in this species, as well as facilitating genetic modifications in the future. Those novel conditions already allowed to better identify the regulons of the central competence regulators in a more relevant way (i.e. through gene deletion) that the previous report based on their overproduction (that could lead to artefactual results). Moreover, oxygen limitation was identified as a key parameter for activation, which make sense regarding the lifestyle of S. aureus during infection. Globally, the manuscript is logically organized and convincing data are provided. Nevertheless, I have a list of suggestions to improve the global presentation and to better highlight the reproducibility of the results.

We would like to thank Reviewer #2 for his/her time to review our manuscript and for the helpful comments and suggestions made. We particularly followed Reviewer #2 advice regarding the presentation of the RNA-seq results as well as a detailed presentation of the reproducibility of our data. Our answers are presented below and the changes or precisions suggested by Reviewer #2 clearly improved our manuscript.

<u>Comments on formal presentation:</u>

1. The current version of the summary is not really informative regarding the key results obtained during this work and could be improved.
We rewrote the summary to better fit our key results.

2. The transcriptomic work and the analysis of SigH, ComK1, and ComK2 regulons is summarized in Table 1 in the main text. This table is not very informative with vague indications (+, +++) and the most important data are presented as supplemental figures. As a large part of manuscript describes those regulons, I would suggest to replace Table 1 by a composite figure that could include Supp Fig. 6 and Supp Fig 10 to show the overlap between the 3 regulons as top panels. For the five functions reported in Table 1, you can generate overlapping circles for the 3 regulons per function for up and down-regulated genes (two bottom panels).
We replaced table 1 by the new Fig 3 following Reviewer #2 suggestions.

3. The reproducibility of data is not fully reported. Indications of technical or biological replicates are missing in most cases. Do the error bars correspond to SEM/SD or confidence intervals? In bar charts, please show individual values as dots. For microscopy, how many cells per replicate were examined? Please indicate that you show a mean value. I would suggest to include all information regarding reproducibility in the figure legends. I would also suggest a homogenous presentation through the whole set of figure legends (including Supp material)
As suggested by Reviewer #2, we included all information regarding reproducibility in the figure legends and tried to homogenize our presentation.

-Fig 1a and b: one representative experiment? How many biological replicates? Between 18 and 24h in the legend, but shown between 16 and 22h in the fig panel. Fig. 1c: 10 replicates, biological or technical? Numbers are mean values +/- SD or SEM?

Fig. 1a and 1b show a representative experiment. The results obtained in this experiment have been confirmed in two other independent experiments.
We corrected the legend regarding the timing.
Fig. 1c shows mean values +/- SD that were calculated based on biological replicates.

-Associated Supp Fig 1: those data are exactly the same as presented in fig 1a and 1b. I do not see the interest to show them again in a supplementary figure.

Supp Fig. 1 shows the correlation between each growth curve and the corresponding evolution of the percentage of competent GFP-expressing cells that do not appear on the same graph in Fig. 1. We followed Reviewer#2 suggestion and removed it.

-Associated Supp Fig 2b: how many replicates? How many cells were examined per replicate?

The experiment presented in Supp Fig. 1b (formerly Supp Fig. 2b) has been replicated 5 times (in independent experiments, biological replicates) for a total of 2500 cells counted.

- Fig. 2: mean values +/- SD? Show individual values. 5 replicates but technical or biological?

In Fig. 2, the numbers were calculated based on at least 5 biological replicates. These numbers show the mean +/- SD. We now show individual values.

- Associated Supp Fig 3a: data are represented as mean +/- SEM, I do not see mean and error bars?

We have done a mistake in the legend. These are not mean values but we rather present all individual points obtained in three independent experiments. We corrected the legend.

- Associated Supp Fig 3b and Supp Fig 4, Fig. 3: Numbers are mean values +/- SD or SEM? Show individual values. Each experiment in each condition has been repeated at least 5 times – this statement is not very clear.

Numbers are mean values +/- SD. We now show individual experiments. We now show individual values.
The sentence has been corrected to make it clearer.

-Fig 4: one representative experiment? How many repetitions?

The experiment presented in Fig. 5 (formerly Fig. 4) is representative of the results consistently observed more than 5 times.

4. Tables are presented as figures: Fig. 1c, Supp Fig. 5, Supp Fig. 7, Supp Fig. 8, and Supp Fig 9 and Supp Fig 11.
For Fig 1c, I would suggest to present the data as a bar chart with values on the top or extract it as a separate table.
For the others, I would name them tables. Please note that for Supp Fig/Table 5, values (ratio?) and color cut-off are not described.

As suggested, we changed Fig. 1c to a bar chart (mean +/- SD) showing individual values.
Based on Reviewer #1 comments, we also created a new Fig. 1d showing transformation efficiencies of additional laboratory isolates or clinical strains. We also made it as a bar chart (mean +/- SD) with individual values.
We finally renamed Supp Fig 5, 7, 8, 9 and 11 as Supp Tables.

5. Please provide a GEO number for the whole set of RNAseq data

We deposited the whole set of RNAseq data in Geo (Gene Expression Omnibus). All the data are now compiled under the GEO submission GSE224932. They are not accessible yet, but we have created a secured token for the reviewers:
To review GEO accession GSE224932:
Go to https://www.ncbi.nlm.nih.gov/geo/query/acc.cgi?acc=GSE224932
Enter token into the box: cnqvqayipxepxsr.

The GEO number is now indicated in the Material and Methods.

<u>Scientific comments:</u>

6. L113-119: DNA transformation with plasmids and chromosomal DNA. I was wondering if the authors have performed trials with linear PCR products including an antibiotic resistance marker and recombination arms (>1 kb). Generally, such donor DNA can lead to high transformation rates. If done, it could be interesting to include those results.

During this project, we had the same idea as Reviewer #2 and tested different kind of donor DNA in order to improve the transformation rates detected. We tested the possibility to transform PCR fragments including an antibiotic marker flanked by 1 to 2kb recombination arms with homology on the recipient cell chromosome. Unfortunately, such setup does not seem to work.

Such result might not be that surprising as transformation with plasmids and chromosomal DNA only works if this exogenous DNA is purified from specific strains with appropriate restriction/modification systems (RN4220 *S. aureus* or IM08B *E. coli* strains for example). This limitation will require some investigation as the ability to transform with PCR products is clearly a huge gain of time.

7. L180-L215: contribution of ComK2 to the whole program of competence development. The authors should better explain why ComK2, that do not control any essential gene required for DNA transformation, is involved in the whole program. They should better explain the overlapping of regulons based on the figure I suggest to replace Table 1. L188-189, please give references.

We agree with Reviewer #2 and better discuss the role of ComK2 as well as the overlapping of regulons (line 281).

We can mention here that we agree that it is surprising that ComK2 is involved in the whole program but not required for DNA transformation. However, these facts have only been demonstrated under specific growth conditions (i.e in CS2 medium). Indeed, we have preliminary data showing that ComK2 might play other roles under different growth conditions. However, we feel uncomfortable to discuss this extensively in this paper as these results will be presented in a future publication.

Moreover, this hypothesis that ComK2 might control different set of genes depending on the environmental conditions is in perfect adequacy with the existence of three central competence regulators and overlapping of their respective regulons. Indeed, each regulator could be involved, activated under specific conditions, by different stresses, allowing an even more complex regulation of the late competence genes expression.

We now provide references line 196

8. L232: SrrAB might control the sigH gene. It should be interesting to show if the promoter of sigH is directly controlled by SrrAB using a PsigH-gfp fusion.

As reviewer #2 suggested, we constructed the P*sigH-gfp* transcriptional fusion. Unfortunately, in a wild type background, *sigH* expression could not be detected. It appears that GFP is not sensitive enough to detect *sigH* low expression. This result is not surprising as the RNAseq study already showed that very few *sigH* mRNAs could be detected.

We do not discuss this in the manuscript yet but if Reviewer #2 thinks that it is important to mention, we will add a comment in the discussion.

9. L249-250 and L285-286: growth in CS2 medium leads to oxygen limitation. Could you give an explanation here or in the discussion (L285-286)? Oxygen limitation probably result in a dramatic change in carbon metabolism which could be more compatible with competence development. A couple of sentences in the discussion should report this and refer to the published work of Cordero et al. (ref 35).

The idea that oxygen limitation occurs during growth in CS2 comes from the original protocol created by Morikawa in 2012. Unfortunately, the important detail in this protocol is lacking from the 2012 paper (Morikawa *et al*., Plos Pathogens, 2012) but is presented in a video associated to another paper published in 2017 in the journal Jove (Cafini et al, 2017. Jove. DOI: 10.3791/55087). The key element in this protocol is that growth in CS2 is performed in a CLOSED 50 mL Falcon tube. Therefore, oxygen limitation does not come from growth in CS2 medium but rather from the fact that oxygen is limited in a closed tube. We already mentioned this important detail in the material and Methods and now discuss this line 302

Regarding the carbon metabolism, we do not think that our conditions lead to the same results as in Cordero et al. Indeed, the Lopez laboratory found that ComK1 was essential for *S. aureus* survival during infection (*Cordero et al., 2022*). They showed that competence induction occurs in response to Reactive Oxygen Species (ROS) and host defenses. These stresses compromise *S. aureus* respiration during infection that needs to switch to fermentation, which is less energetically efficient. Induction of ComK1 would then activate the expression of genes involved in the central carbon metabolism, leading to an increase in the glycolytic flux and therefore *S. aureus* survival during infection. In such conditions, genes involved in the carbon metabolism are therefore part of the ComK1 regulon and should be considered as late competence genes (and probably not involved in the early steps of competence development). We do not find such genes in our study under the control of ComK1.

However, we now mention in the discussion (line 311) that drastic changes in the carbon metabolism could be interesting to investigate in the context of competence development.

10. L281: transformation protocol, dilutions were incubated with shaking. Could you explain why cultures were agitated, knowing that microaerobic conditions are required for optimal competence activation. Is it for GFP maturation?
L465, why agitation during the transformation assay?

Our optimized protocol is based on the protocol published by Morikawa in 2012 where incubation was performed with shaking. Since the tubes in which the cultures are performed are closed, the shaking might favor the consumption of the dissolved oxygen, which leads to microaerobic conditions as the atmospheric oxygen is limited.

We do not think that shaking is important for GFP maturation since we can also perform the same king of experiment in true anaerobic conditions. The cells sampled for flow cytometry are finally exposed to atmospheric oxygen which we think is enough to allow the GFP detection.

The transformation assay is also based on the protocol published by Morikawa. It would be interesting to test if this is important to perform natural transformation.

<u>Minor comments/corrections:</u>

11. Page numbers are missing.
We have now included page numbers.

12. In all numerical values, replace the comma by a dot in figures and text (e.g. L115, L117, …)
Done

13. Check carefully gene and protein names for capitals and italics (e.g. L147, PcomF; …). For promoters, the gene name in italics is underscored.
All gene and protein names have been corrected

14. The use of "would" is not always appropriate. Please check
We checked and corrected when needed all the occurrences of the word "would".

REVIEWERS' COMMENTS:

Reviewer #1 (Remarks to the Author):

The authors have adequately addressed all of my previous questions, and I only have one final minor comment. In my previous report, I mentioned that the use of "natural" competence may be inappropriate. Although the authors changed it to "natural transformation," I still believe that this term is incorrect. The authors induce competence/transformation, so I suggest using the term "induced competence" instead. This term seems more appropriate until the mechanism of competence is fully understood in S. aureus.

Reviewer #2 (Remarks to the Author):

The manuscript by Feng et al. has been significantly improved in its content by bringing new data and in its global presentation (statistics and figures). The authors appropriately answer to most of my requests. Nevertheless, I have still a short list below of minor comments/corrections.

L38: "the regulon of each central regulator"
L82: change 1,6% in 1.6%
L86-85: this is a very long sentence that needs to be rephrased for clarity
L117: "under laboratory conditions, the N315…"
L134: "two-hour delay in growth" – I do not see any growth delay in Fig 1a – this could be just removed if not shown
L135: "…diluted culture (Fig. 1b)."
L137: "… stationary phase (Fig. 1a and Fig. 1b)."
L154: "and PcomF" F in italics
L178-179: "… Supplementary Table 4)". This is a table and not a figure
L210-211: 399 genes were inhibited – please indicate the fold change (> 2 fold)
L211: comk2, k in capital letter
L221-227: besides the fact that only 50% of the population developed competence, you collect your cells at a defined time point during growth. This time point could not be appropriate to observe the effect on the exoproteome. This could be added as a possible explanation.
L362: "induced (a) or repressed (b) (> 2 fold)"
L368: "late competence genes (> 2 fold)"
Fig. 1 panel B – replace coma by dot in all numbers
Supplementary Fig 1 title: change "identical" by "similar"
Supplementary Fig 2 panel A and corresponding legend: each color represent data from one independent experiment. There are many dots with the same color for a defined OD. I guess that each individual experiment includes technical replicates – please give this information in the legend.
Supplementary Fig. 4: this is not a figure but a table! So, Supplementary Table 1. Please add a footnote to define the color code (green, blue, and orange) and the red color of some numbers. Please explain how numbers were calculated in the footnote. I guess that those are ratio between WT and mutant normalized reads.
Supplementary Table 1: change "this Figure is…" in "This table is…"
Supplementary Table 3: change "this Figure is…" in "This table is…"

Rebuttal letter

18th April 2023

Here is a point-by-point answer to the reviewers' comments. Again, we thank them for their time and comments/suggestions that really helped the readability and general strength of our paper.

Reviewer #1 (Remarks to the Author):

The authors have adequately addressed all of my previous questions, and I only have one final minor comment. In my previous report, I mentioned that the use of "natural" competence may be inappropriate. Although the authors changed it to "natural transformation," I still believe that this term is incorrect. The authors induce competence/transformation, so I suggest using the term "induced competence" instead. This term seems more appropriate until the mechanism of competence is fully understood in S. aureus.

We have removed the term “natural” in front of competence in the title and in general in the text. We now just mention competence (without any adjective) and genetic transformation (which is the name of the process).

Reviewer #2 (Remarks to the Author):

The manuscript by Feng et al. has been significantly improved in its content by bringing new data and in its global presentation (statistics and figures). The authors appropriately answer to most of my requests. Nevertheless, I have still a short list below of minor comments/corrections.

L38: "the regulon of each central regulator"
Done (new line 29)

L82: change 1,6% in 1.6%
Done (new line 63)

L86-85: this is a very long sentence that needs to be rephrased for clarity
Done (new lines 67-70)

L117: "under laboratory conditions, the N315…"
Done (new line 97)

L134: "two-hour delay in growth" – I do not see any growth delay in Fig 1a – this could be just removed if not shown
We are sorry but we do not understand the problem with this sentence. The growth delay between successively diluted cultures seems clear. The confusion might come from our choice of words. To better explain this delay, we added a sentence lines 115-117: "Indeed, the 10^{-5} dilution needs more time to reach stationary phase than the 10^{-4} dilution, which needs more time than the 10^{-3} dilution".
As the growth delay seems clear and because the paragraph is articulated around this notion, with the induction of competence being delayed accordingly with growth, we do not intend to remove this sentence.

L135: "…diluted culture (Fig. 1b)."
Done (new line 118)

L137: "… stationary phase (Fig. 1a and Fig. 1b)."
Done (new line 119)

L154: "and PcomF" F in italics
Done (new line 137)

L178-179: "… Supplementary Table 4)". This is a table and not a figure
We changed the Supplementary Fig. 4 into the Supplementary Table 1.

L210-211: 399 genes were inhibited – please indicate the fold change (> 2 fold)
Done (new line 194)

L211: comk2, k in capital letter
Done (new line 194)

L221-227: besides the fact that only 50% of the population developed competence, you collect your cells at a defined time point during growth. This time point could not be

appropriate to observe the effect on the exoproteome. This could be added as a possible explanation.
Done (new lines 210-211)

L362: “induced (a) or repressed (b) (> 2 fold)”
Done (new line 346)

L368: “late competence genes (> 2 fold)”
Done (new line 352)

Fig. 1 panel B – replace coma by dot in all numbers
Done

Supplementary Fig 1 title: change “identical” by “similar”
Done

Supplementary Fig 2 panel A and corresponding legend: each color represent data from one independent experiment. There are many dots with the same color for a defined OD. I guess that each individual experiment includes technical replicates – please give this information in the legend.
Done. In fact, in the Supplementary Fig. 2a, the same experiment as in Fig. 1a and 1b has been conducted and repeated three times. Therefore, the dots with the same color present at a defined OD come from different dilutions in the same experiment. We added a sentence in the legend to better explain this.

Supplementary Fig. 4: this is not a figure but a table! So, Supplementary Table 1. Please add a footnote to define the color code (green, blue, and orange) and the red color of some numbers. Please explain how numbers were calculated in the footnote. I guess that those are ratio between WT and mutant normalized reads.
We changed the Supplementary Fig. 4 into the Supplementary Table 1.
We also now explain the color code and the numbers were calculated.

Supplementary Table 1: change “this Figure is…” in “This table is…”
Supplementary Table 3: change “this Figure is…” in “This table is…”
Done
